# Physical Heterogeneity Increases Biofilm Resource Use and Its Molecular Diversity in Stream Mesocosms

**DOI:** 10.1371/journal.pone.0009988

**Published:** 2010-04-01

**Authors:** Gabriel Singer, Katharina Besemer, Philippe Schmitt-Kopplin, Iris Hödl, Tom J. Battin

**Affiliations:** 1 Department of Freshwater Ecology, University of Vienna, Vienna, Austria; 2 WasserKluster Lunz GmbH, Lunz am See, Austria; 3 Institute of Ecological Chemistry, Helmholtz Zentrum München, German Research Center for Environmental Health, Neuherberg, Germany; University of Oxford, United Kingdom

## Abstract

**Background:**

Evidence increasingly shows that stream ecosystems greatly contribute to global carbon fluxes. This involves a tight coupling between biofilms, the dominant form of microbial life in streams, and dissolved organic carbon (DOC), a very significant pool of organic carbon on Earth. Yet, the interactions between microbial biodiversity and the molecular diversity of resource use are poorly understood.

**Methodology/Principal Findings:**

Using six 40-m-long streamside flumes, we created a gradient of streambed landscapes with increasing spatial flow heterogeneity to assess how physical heterogeneity, inherent to streams, affects biofilm diversity and DOC use. We determined bacterial biodiversity in all six landscapes using 16S-rRNA fingerprinting and measured carbon uptake from glucose and DOC experimentally injected to all six flumes. The diversity of DOC molecules removed from the water was determined from ultrahigh-resolution Fourier Transform Ion Cyclotron Resonance mass spectrometry (FTICR-MS). Bacterial beta diversity, glucose and DOC uptake, and the molecular diversity of DOC use all increased with increasing flow heterogeneity. Causal modeling and path analyses of the experimental data revealed that the uptake of glucose was largely driven by physical processes related to flow heterogeneity, whereas biodiversity effects, such as complementarity, most likely contributed to the enhanced uptake of putatively recalcitrant DOC compounds in the streambeds with higher flow heterogeneity.

**Conclusions/Significance:**

Our results suggest biophysical mechanisms, including hydrodynamics and microbial complementarity effects, through which physical heterogeneity induces changes of resource use and carbon fluxes in streams. These findings highlight the importance of fine-scale streambed heterogeneity for microbial biodiversity and ecosystem functioning in streams, where homogenization and loss of habitats increasingly reduce biodiversity.

## Introduction

A growing body of reviews and meta-analyses has recently highlighted the functional consequences of biodiversity for ecosystem functioning [1], [2], [3], [4]. Collectively, these studies suggest biodiversity effects on ecosystem functioning, although highly variable in strength, across most study systems. Experiments typically manipulate biodiversity by randomly generating an array of species combinations from a species pool in homogeneous systems with little physical, chemical and biological structure–an approach that is frequently and increasingly criticized because of its lack of realism [5], [6]). Theory predicts environmental heterogeneity to allow expression of niche differences and thereby biodiversity effects [7], [8] and empirical work on algae [9] plants [10], [11] and macrofauna [12], [13], [14] increasingly support this prediction. Microorganisms, however, the most diverse organisms on Earth [15], have remained largely exempt from this research [16]. Microorganisms are tightly linked with biogeochemical cycles and ecosystem functions at all scales [17]. Key to this fundamental link is dissolved organic carbon (DOC), a large pool of reduced carbon [18], which, depending on its bioreactivity, is taken up by heterotrophic microbes thereby mediating trophic transfer and carbon fluxes [19], [20]. In most freshwater ecosystems DOC is predominantly of terrestrial origin, conventionally classified as recalcitrant and yet fueling the net heterotrophy of these ecosystems [21]. The advent of novel ultrahigh-resolution mass spectrometry is now increasingly unveiling the molecular complexity of DOC in various aquatic ecosystems [22], [23], [24], [25], [26]. However, the link between molecular diversity of DOC and microbial biodiversity remains elusive (e.g., [24], [27]) despite its potential consequences for ecosystem functioning.

In this paper, we report the results of an experiment designed to test the hypothesis that fine-scale physical heterogeneity in streamside flumes affects bacterial biodiversity and DOC resource use by benthic biofilms. Biofilms are matrix-enclosed and attached communities that dominate microbial life in streams where they can control key ecosystem functions [28]. We experimented with 40-m-long streamside flumes and constructed streambed landscapes with varying spatial heterogeneity. Bedforms, typical for low-submergence headwater streams, induced spatial gradients of velocity and streambed landscapes of increasing flow heterogeneity (i.e., spatial variation of flow conditions); flume-average velocities and residence times were kept nearly identical across flumes. We studied how spatially heterogeneous flow offers opportunities for benthic biofilms to develop into diverse communities from the same regional species pool (i.e., the inoculum in the streamwater) in microhabitats along the bedforms, and how flow heterogeneity and biofilm diversity affect DOC removal and its diversity. We challenged biofilms with the hydrophobic fraction of DOC leached from riparian vegetation since this fraction, rather than the hydrophilic fraction, has typically reduced bioavailability [29], [30]. Ultrahigh-resolution mass spectroscopy served to describe the molecular diversity of DOC removed from the water and molecular fingerprinting based on 16S rRNA was used to infer microbial biodiversity.

## Materials and Methods

### Experimental setup

**Figure 1:**
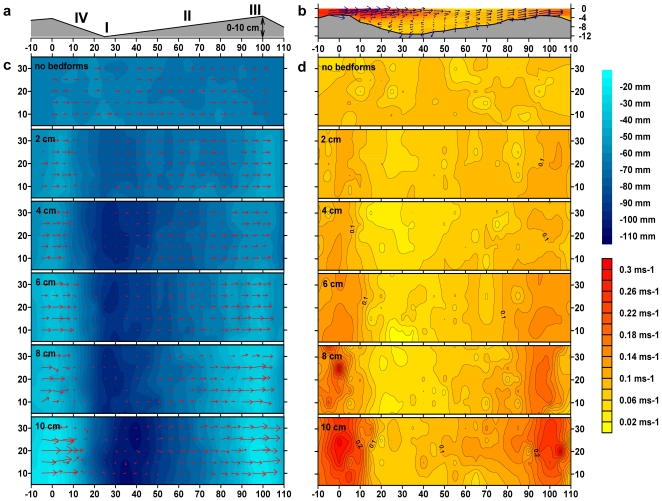
Constructed landscapes differing in flow heterogeneity. Graphs describe shape of and hydrodynamic conditions over approximately one bedform (38 per flume). X-axis represents longitudinal flume dimension (equivalent to main flow direction, total flume length 40 m) with distances given in cm. (**a**) Side-view of impermeable 1-m-long bedform lining the bottom of the mesocosm. Bedforms of variable height (2–10 cm) were used to create the heterogeneity gradient. I, II, III and IV show the positions (“microhabitats”) of ceramic coupons used for determination of biomass and T-RFLP. (**b**) A representative vertical cross section along the thalweg in the flume with 8 cm bedforms showing water velocity conditions above a single bedform. Y-axis represents depth below water surface (in cm), contours show length of velocity vector, blue arrows show amount and direction of current. (**c**) and (**d**) show contour plots of depth (c, blue) and velocity (d, length of 3-D vector R_xyz_, yellow-orange) above a single bedform in each of the 6 heterogeneity treatments, Y-axis represents lateral flume dimension (distances in cm, total flume width 0.4 m) and red arrows show amount and direction of current.

Experiments were conducted in streamside flumes (length: 40 m, width: 0.4 m). Five flumes contained 38 triangular dune-shaped bedforms (Figure 1a), respectively, with a height of 2, 4, 6, 8 and 10 cm. Bedforms were periodically installed to create landscapes of differing velocity variance (i.e. flow heterogeneity). One flume without bedforms served as control. Bedforms were impermeable and covered with a monolayer of natural graded gravel (<4 cm diameter) as substrate for biofilm growth. Pore space was thus minimized to reduce confounding effects from surface-subsurface hydrodynamic exchange and related processing rates. All flumes received the same raw stream water (Oberer Seebach, Austria) with the same constant discharge (2.25 L s^−1^). The resulting flow may be identified as shallow rough-bed flow typical for headwater streams. The flumes were continuously fed in a once-through mode to assure identical water chemistry and microbial inoculum. A header tank and tilting weirs ensured steady and spatially uniform flow. Nearly identical average flume-level velocities were achieved by adjusting flume slopes individually with repeated measurements of mean velocity by slug additions of NaCl-solutions [31]. We thus created flow environments of differing velocity variance (i.e. spatial flow heterogeneity) but identical mean water velocities.

### Flow heterogeneity

Acoustic Doppler Velocimetry (Vectrino® Nortek, 4-beam side-looking probe, 50 Hz for 1 min, time series of n = 3000) described the 3-D flow fields and served to derive heterogeneity of flow as its spatial variation. In each flume, velocity was mapped over one entire bedform (∼5 mm above substrate) at nodes of a 5×5 cm grid. For each node, we determined depth, mean length of the 3-dimensional velocity vector 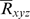, turbulent kinetic energy TKE, and turbulence intensity TI (see Text S1). 80 measurements per flume yielded reliable estimates for description of the average flow environment and its spatial variation. *SD*_Rxyz_, the standard deviation of *R*_xyz_ is the central independent variable and a surrogate for flow heterogeneity.

### Biofilm communities

Sterile unglazed ceramic coupons (1×2 cm) served as substratum for biofilm growth [32]. In each flume, coupons were sampled from 4 distinct microhabitats (upstream side, crest and downstream side of each bedform, and the trough between two bedforms, all along the thalweg, see Figure 1a), along triplicate bedforms (72 coupons per sampling date and parameter) within a restricted flume segment for microbial biomass (bacterial biomass and chlorophyll-*a*) and for bacterial community composition. Coupons were sampled four times over a growth period of 4–5 weeks during summer season. Biofilms reached stationary growth after approximately 3–4 weeks. Coupons from triplicate bedforms were processed as composite samples for bacterial community composition. To determine microbial biomass SYTOX-stained bacteria were counted and sized using a Cell-Lab-Quanta (Beckman Coulter). Chlorophyll-*a* was assayed as described elsewhere [32]. Microscopy was used to describe biofilm architecture at several spatial scales.

### Terminal-restriction fragment length polymorphism

DNA from biofilm communities was extracted and purified with the UltraClean Soil DNA Isolation kit (MoBio Carlsbad, Calif.). Non-colonized ceramic coupons served as negative controls. The fluorescently labeled primers used for PCR of the 16S rRNA gene were FAM labeled 27F and JOE-labeled 1492R (Thermo Electron, Germany) [4]. PCR was performed as described elsewhere [33]. PCR-products were cleaned using gel electrophoresis and the QIAquick Gel Extraction kit (Qiagen). Restriction digests were done as described earlier [33], using approximately 300 ng DNA and the enzyme HhaI. The products were desalted by gel filtration using MultiScreen-HV 96-well plates (Millipore), loaded with Sephadex G-50 (Sigma). The dried product was re-suspended in 10 µl HIDI formamide and 0.5 µl size marker GS2500 Rox (Applied Biosystems), denatured at 95°C and immediately placed on ice. DNA fragments containing the fluorescently labelled forward primer were separated in a Capillary Sequencer 3130 Xl (Applied Biosystems), and electropherograms were analyzed with the GeneMapper software. Restriction fragments smaller than 30 bp and larger than 900 bp were excluded from further analysis to avoid detection of primers and uncertainties of size determination. Peaks >2% of maximum peak height were clearly distinguishable from background noise. The relative contribution of the respective operational taxonomic units (OTUs) to total community was estimated as peak height divided by the cumulative peak height of the given sample [34], [35]; TRFLP analysis can provide reproducible quantitative results [34], [35], [36]. Fragments containing the forward and the reverse primer were analyzed separately. T-RFLP patterns produced with the forward primer showed generally more heterogeneity in restriction fragment size than the corresponding T-RFLP patterns containing the reverse primer. Results in this study refer to the forward fragments because of their higher information content.

### Biodiversity partitioning and community composition

As the use of diversity indices is limited for molecular fingerprint data [33], [34], we employed various diversity indices of the Hill-family [35], namely richness, the Shannon entropy and the Gini-Simpson coefficient, which differ in their sensitivity towards rare species. Diversity partitioning was done according to Jost [36]. In addition, we used the average Bray-Curtis distance between samples from one flume at a given time as a complementing measure for beta diversity. Regional diversity was also decomposed into the 2 independent orthogonal components richness and evenness following the evenness definition of Hill [35], [36], [37]. This decomposition followed by separate analysis seems important as evenness and richness may not necessarily correlate positively and may not lead to the same ecosystem process response (e.g. [37], [38]. Pielou's classical Shannon evenness definition as *ln(^q = 1^D)/ln(S)* was included in our analyses because of its traditionally widespread use in ecology. Molecular fingerprinting has limited ability to detect numerically minor taxa [34], and we therefore repeated the analysis using reduced datasets with taxa common to all flumes or all microhabitats. To oppose effects of diversity components to effects of species identity (“selection” or “sampling” in the context of the biodiversity-ecosystem function debate), we described similarity among flumes with regard to community composition at flume level (gamma) by computing Bray-Curtis dissimilarity matrices between flumes. See Text S1 for rationale and details of computation of these various metrics.

### Glucose and DOC uptake

We produced several hundreds of liters of filtered cold-leaf-leachate (48 h) from riparian vegetation (*Petasites* sp., *Phalaris* sp., *Mentha* sp., *Veratrum* sp., *Cirsium oleraceum)* to study dynamics of complex DOC. This DOC from riparian vegetation mimics an important terrestrial subsidy, which can generate substantial pulses of DOC in headwaters [39]. Mass uptake of glucose (as a highly labile control substrate) and DOC was measured by separately injecting the respective solutions into the flumes and monitoring concentration loss in recirculating systems over 6–8 hours on multiple occasions. Recirculation was done to increase interaction time of solutes with the biofilm; devices associated with recirculation were free of biofilm. Experiments were performed with no biofilm and with 24 d old biofilm for DOC; and with no biofilm and with 12, 18 and 23 d old biofilm for glucose, respectively. Starting concentrations were 600–900 nmol L^−1^ and 8 mg L^−1^ for glucose and DOC, respectively. Water samples were collected at the flume outlets, filtered and frozen immediately before analysis. Glucose was analysed with HPLC–PAD (Dionex ICS3000) using a CarboPac PA20 column (30°C, 20 mM NaOH eluent, flow rate 0.5 mL min^−1^). DOC concentration was measured using a SIEVERS (Ionics Instruments) and a Shimadzu TOC-V_CPH/CPN_. Mass transfer coefficients (uptake velocity v*_f_*, units length time^−1^) for glucose and bulk DOC were calculated from log-linear fits of concentration decline in time (see Text S1).

### DOC composition

DOC (2 L water samples) from the header tank (inflow) and from each flume after recirculation (residual) was extracted using XAD-8 resin [40]. Fourier-transform-ion cyclotron-mass spectrometry (FTICR-MS) was performed in ESI(−) mode on a Bruker (Bremen, Germany) 12 T APEX-Qe FTICR-MS equipped with an APOLLO-II ionization source [41]. Mass spectra (750 accumulated scans) were acquired with a time domain of 1 megaword over a mass range of 100–2000 m/z. Peak lists were exported at a signal-to-noise-ratio of 1 and elemental formulae were calculated for a maximum composition of C_100_O_80_N_5_S. See Text S1 for calibration details, validity checks and tolerance limits. To exclude sensitivity and contamination artifacts all data analyses were conservatively limited to compounds positively identified in the inflow and in all residual samples (361 individual peaks). 276 peaks had reliably assigned mass formulae within tolerance limits. DOC-diversity was calculated from relative peak intensities and compound-specific mass transfer coefficients were computed from peak intensity ratios of inflow and residual. Multiple linear regression and hierarchical partitioning [42] identified a subset of predictors repeatedly explaining most of the variance of compound-specific mass transfer in individual flumes. Reduced models were used to investigate gradients among flumes by relating unstandardized slopes of the regression models to environmental heterogeneity (*SD*_Rxyz_). See Text S1 for details of data analysis.

### Data synthesis

We hypothesized biodiversity components and community composition as explanatory links between environmental heterogeneity and resource use. Because of the multivariate nature of most of the involved entities, we used causal modeling on dissimilarity matrices calculated from subsets of appropriate variables [43] to explore hypothesized causal links between the metavariables flow heterogeneity, structural and compositional biofilm diversity, community composition, resource use diversity and ecosystem functions. Mantel and partial Mantel (controlling for environmental heterogeneity) tests with full-randomized distributions (6! permutations) were used to test for associations. Mantel statistics do not have to be large to be statistically significant. They are related to correlation coefficients and we calculated path coefficients to test hypothesized causal relationships and facilitate visual representation. Significance of path coefficients was calculated by randomization of all involved matrices using 10^5^ permutations. All calculations were done in R [44], using packages vegan and sem.

## Results

### Physical heterogeneity

High-resolution mapping of 3D-flow velocimetry with Acoustic Doppler Velocimetry revealed flow environments typically for bedforms (Figure 1). Flow fields became more variable as bedform height (2, 4, 6, 8 and 10 cm; one flume without bedforms served as control) increased. Flow velocity generally accelerated along the upstream side and decelerated along the downstream side of the bedforms. Turbulent kinetic energy increased accordingly and was highest downstream of the bedform crest, whereas turbulence intensity was highest between consecutive bedforms, where wake-induced eddies formed. These fine-scale changes over multiple bedforms of the same dimension caused spatial heterogeneity of flow, expressed as the standard deviation of the average 3-dimensional flow velocity vector, to increase from 1.6 cm s^−1^ to 6.3 cm s^−1^ across all six flumes (Figure S1). Individually adjusting the slope of each flume, we achieved to keep nearly identical mean flow velocity (7.6±0.4 cm s^−1^ which translates to a residence time of 8.8 min) and mean water depth (6.7±0.4 cm) among flumes (Table S1). Background DOC and nutrient concentrations in the streamwater were almost identical across all six flumes (Table S2). Given this setting and the fact that all flumes were continuously fed from the same water reservoir, we are confident that our experimental design isolated physical spatial heterogeneity as the major factor.

### Microbial biomass distribution and biofilm architecture

**Figure 2:**
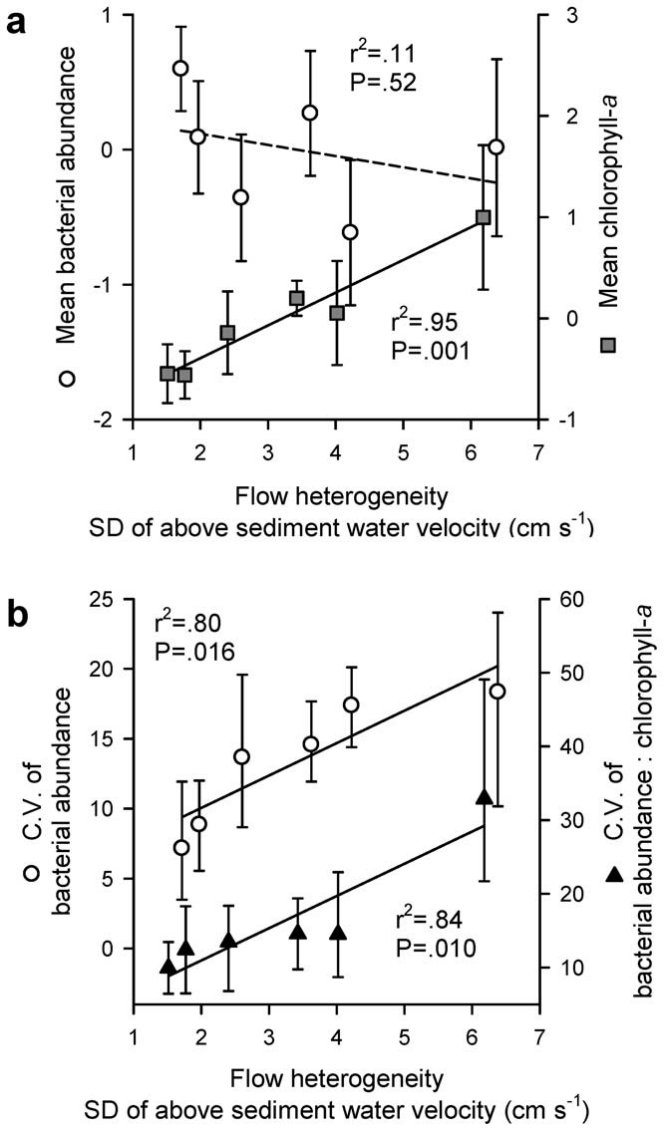
Microbial biofilm biomass. (**a**) Flow heterogeneity (SD_Rxyz_) versus mean z-standardised bacterial abundance and chlorophyll-*a* (error bars represent 95% confidence intervals, sample size n = 12 per flume); and (**b**) flow heterogeneity versus the average coefficient of variation of bacterial abundance and the bacterial abundance to chlorophyll-*a* ratio (error bars represent 95% confidence intervals generated by bootstrapping, sample size n = 4 per flume, see Methods).

**Figure 3:**
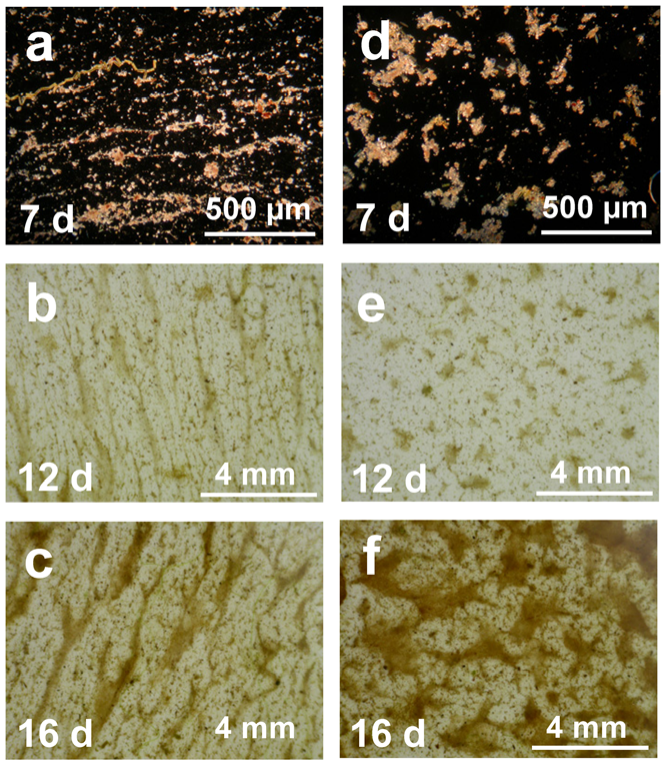
Microbial biofilm structure. Microscopic darkfield and orthophotographic images of biofilms from the crest (**a–c**) and the trough (**d–f**) between 2 consecutive bedforms. Arrows on indicate main flow direction, numbers indicate biofilm age in days.

Chlorophyll-*a* concentration correlated with flow heterogeneity (as the standard deviation of the water velocity above sediment) whereas bacterial abundance did not (Figure 2). The spatial coefficients of variation (CV) of chlorophyll-*a* and bacterial abundance, computed from four repeatedly sampled microhabitats along the bedforms, clearly increased with flow heterogeneity (Figure 2). These patterns suggest structural differentiation of the biofilms with increasingly higher bedforms. This was in fact supported by microscopic analysis of biofilms from two microhabitats with distinct hydrodynamics — the crest and the trough (Figure 3). Around the bedform crest, biofilms developed directed architectures with filamentous streamers floating in the water, whereas in deeper and low-shear microhabitats between bedforms, multidirectional flow generated non-directed and connecting microcolonies.

### Bacterial biodiversity

**Figure 4:**
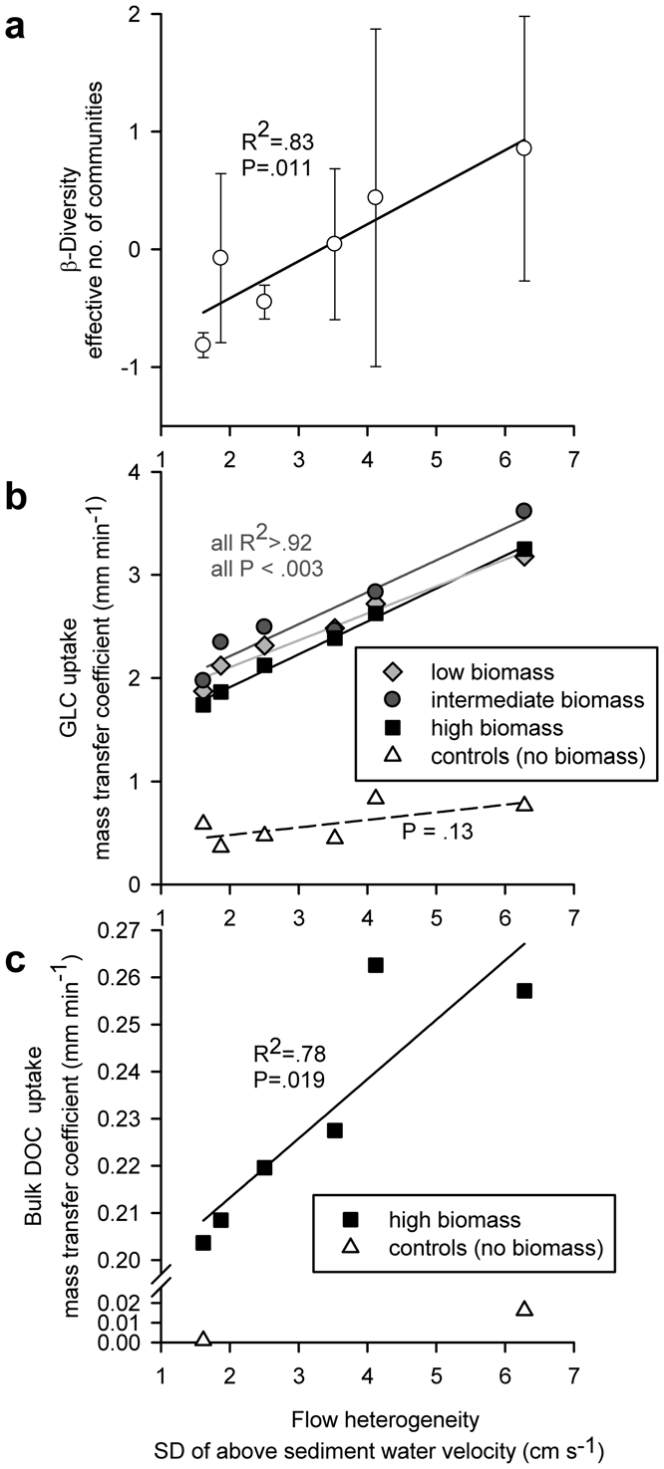
Relationship between flow heterogeneity and bacterial biodiversity and carbon mass transfer. The standard deviation of *R*_xyz_ (i.e. the length of the 3-dimensional vector of velocity) explains (**a**) b-diversity, as the effective number of communities using an Hill exponent q = 1 (data pooled from multiple sampling dates and z-standardised, sample size n = 4 per flume on each of 4 dates); (**b**) glucose mass transfer coefficients at various biofilm growth states; and (**c**) bulk DOC mass transfer coefficients. Triangles indicate controls with no biofilms in (b) and (c).

T-RFLP analyses identified a total of 76 bacterial operational taxonomic units (OTUs) in the biofilm samples in all flumes with an average of 26±13 (mean±SD) per flume. Total bacterial diversity at flume-scale (regional or gamma diversity) was partitioned into the two orthogonal independent components alpha (average diversity within microhabitats) and beta (among microhabitats) diversity, and into richness and evenness, respectively (see Methods). We employed various diversity indices of the Hill-family [35], namely richness, the Shannon entropy and the Gini-Simpson coefficient, which differ in their sensitivity towards rare species. We found beta diversity to significantly increase with flow heterogeneity, while neither alpha nor gamma (flume-level diversity) diversity (nor any evenness measure) did (Fig. 4a, SI Table S6). Interestingly, this pattern remained unchanged upon conservative re-analysis that excluded rare OTUs prone to sampling error by T-RFLP [45]. In fact, by using only OTUs found in all microhabitats (i.e., samples) at each date, we reduced the analysis to a conservative investigation of pure evenness effects among widespread and common taxa. This approach revealed patterns of beta diversity that were even stronger with higher slopes when plotted against flow heterogeneity and higher coefficients of determination. Still no significant effects of flow heterogeneity on gamma diversity or gamma evenness could be found. The stability of these diversity patterns reduces the likelihood of artefacts often inherent to T- RFLP analysis. Similarly, mean Bray-Curtis distances among the four microhabitats along the bedforms, another robust measure for beta diversity, significantly increased with flow heterogeneity (r^2^ = 0.89, P = 0.005). Thus, we could identify a relatively small but stable beta-component that is clearly related to the hydrodynamic microenvironment and largely relies on relative abundance patterns of frequent OTUs.

### Resource use

To explore the possible consequences of spatial flow heterogeneity and related biodiversity patterns for resource use as an ecosystem process, we first measured the uptake of experimentally added monomeric glucose and then of a complex DOC mixture by mature biofilms typical for baseflow in headwaters. We extracted DOC from riparian vegetation to simulate a major carbon input to streams during leaf litter fall in autumn [39], [46]. Leaf packs accumulating on the streambed release large amounts of DOC (including carbohydrates) [46], which become potentially available to the benthic biofilms. In contrast to the highly labile glucose, the hydrophilic component of the leachate, generally assumed to be relatively recalcitrant to microbial uptake [29], [30] challenged biofilms.

We computed mass transfer coefficients (*v*_f_, see Methods) of glucose, bulk DOC and individual DOC-compounds, which are equivalent to the average vertical velocity at which solutes are removed from the water column, and which represent a measure of uptake efficiency relative to availability. *v*_f_ is independent from scaling effects of velocity and flume depth; its product with concentration represents the average flux of a solute to the streambed. We found *v*_f_ of glucose (v_f-Glucose_) and bulk DOC (v_f-DOC_) significantly increased with increasing flow heterogeneity (Figure 4b, c); controls without biofilms confirmed that the observed v_f-Glucose_ and v_f-DOC_ v_f_ were attributable to microbial activity.

**Figure 5:**
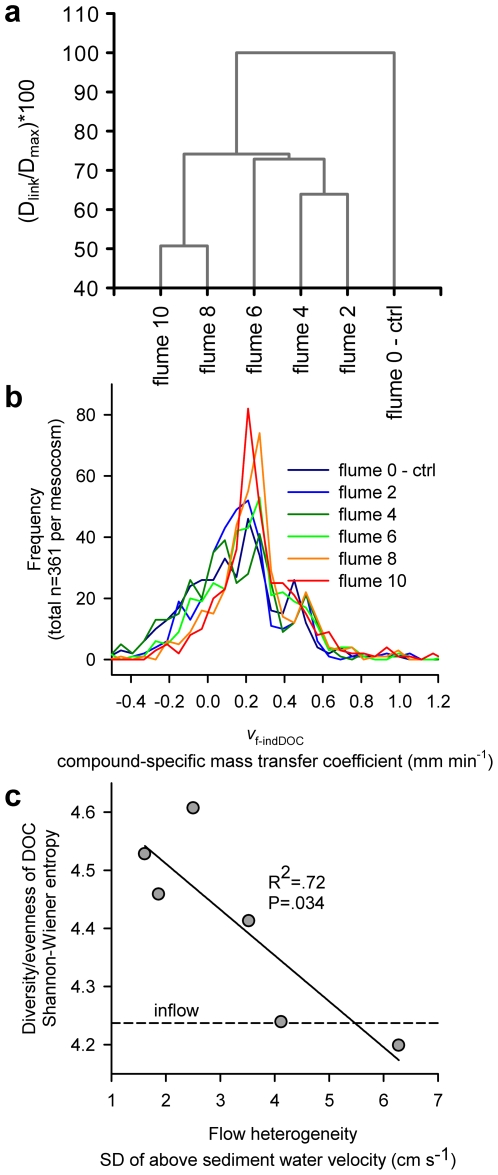
DOC resource use. (**a**) Dendrogram (UPGMA clustering algorithm) showing similarity of resource use distribution among all 6 flumes with increasing flow heterogeneity (height of bedforms in cm). An Euclidean distance matrix was calculated from normalized mass transfer coefficients of 361 DOC compounds present in all residual (after recirculation) and inflow samples. (**b**) Frequency distributions of compound specific mass transfer coefficients (n = 361) in all 6 flumes. (**c**) Shannon-Wiener diversity/evenness of the molecular composition of the residual DOC pool. Note the similar evenness of the inflow (dashed line) and residual DOC pools in the high-heterogeneity landscapes. The decreasing trend indicates a gradual additional uptake of compounds of low relative concentration in the inflow with increasing heterogeneity (analysis on 361 individual compounds identified from all samples).

To test whether the observed increase in *v*_f-DOC_ with flow heterogeneity was paralleled by compositional shifts in the DOC removed by the biofilms, we analyzed the molecular composition of the hydrophobic DOC fraction (from solid-phase extraction) using ultrahigh-resolution Fourier Transform Ion Cyclotron Resonance Mass Spectrometry (FTICR-MS, see Methods). Data analysis remained restricted to those compounds that were identified in both the inflow and all residual (after recirculation) samples to exclude compounds that were likely produced within the flumes (e.g., algal exudates). We hypothesized that without effects of flow heterogeneity nor of compound-specific characteristics of DOC other than concentration, mass transfer coefficients would be equal for all DOC compounds. Comparative analyses of the molecular composition of the inflow and the residual (i.e., after recirculation) DOC pools in each flume revealed increased similarity of resource use (i.e. distribution of uptake among various DOC compounds) among flumes with similar heterogeneity (Figure 5a). Compound-specific mass transfer coefficients (*v*_f-indDOC_, 361 compounds) exhibited less variation at higher flow heterogeneity, indicating more even (i.e., uniformly distributed) consumption across all compounds of the DOC mixture (Figure 5b). This would translate into decreasing metabolic specialization as flow heterogeneity increases. Evenness patterns of the molecular composition of both the inflow and residual DOC pools (Figure 5c) indicate that resource use was limited to the more abundant compounds in less heterogeneous flow, while it gradually included less abundant compounds as flow heterogeneity increased.

Furthermore, hierarchical partitioning analysis (see Methods and Text S1) consistently identified the relative availability in the inflow DOC pool (computed as the relative FTICR-MS peak intensity, *rI*_inflow_) as a key predictor of compound-specific mass transfer coefficients (*v*_f-indDOC_) in individual flumes; followed by the O∶C ratio, H∶C ratio and aromaticity (Tables S4 and S5, Fig. S2). These results show a clear effect of compound chemistry on mass transfer. Compounds of higher relative availability (as *rI*_inflow_) are preferentially removed from the stream water – decoupled from their absolute uptake proportional to concentration (implicit to mass transfer, *v*_f_). These results also suggest that more oxygenated compounds are removed more readily from the water. Specifically, regression coefficients of the O∶C ratio, H∶C ratio and aromaticity (Table S5) suggest consumption of carbohydrates and oxygen-rich aromatics, which are also likely compounds of the fresh leaf leachate. Other predictors, such as the presence of nitrogen atoms and molecule size, did not influence *v*_f-indDOC_ in individual flumes.

In support of the evenness pattern (Figure 5c), the strength of the relationship (expressed as the slope of the regression model) between *rI*_inflow_ and *v*_f-indDOC_ in individual flume models significantly (r^2^ = 0.71, P<0.05) decreased with increasing flow heterogeneity. Similarly, negative relationships between slopes of predictors (or predicted effect sizes) of individual flume models and flow heterogeneity were found for O∶C, aromaticity and nitrogen (Table S5). In the case of O∶C ratios, gradients across flumes with increasing flow heterogeneity were weak. Nitrogen proved to be a good predictor across flumes while its effect remained negligible in the within-flume regressions. A reduced model built on *rI*_inflow_ and nitrogen as the predictors best explaining the heterogeneity gradient had significantly lower prediction quality (indicated by r^2^) with higher heterogeneity (r^2^ = 0.70, P<0.05, Table S5). These results indicate increasing independence of an individual compound mass transfer from controlling factors — either availability (i.e., *rI*_inflow_) or intrinsic chemical properties — at higher flow heterogeneity. Hence, we further used the model coefficients as a proxy for resource use diversity.

The typical way of qualitatively visualizing ultrahigh-resolution spectra are Van Krevelen diagrams showing compounds in a chemical space defined by H∶C versus O∶C [41], [47]. While this approach makes exhaustive and less restrictive use of the wealth of data generated by FTICR-MS, it is potentially compromised by sensitivity issues and contamination effects. Van Krevelen diagrams of inflow and residual DOC pools in the control flume and the flume with highest bedforms (Figure S2) allowed to recognize the preferential removal of O-rich and H-poor compounds as well as the effect of aromaticity and nitrogen. Van Krevelen diagrams were thus able to confirm our extensive quantitative analysis.

### Relating flow heterogeneity to biodiversity and resource use

Our experiments revealed a significant positive relationship between bacterial biodiversity and the diversity of DOC compounds removed from the water. This is supported by the slope of the relationship between *v*_f-indDOC_ and *rI*_inflow_ (as a proxy for resource use diversity), which correlates better with bacterial beta diversity than with flow heterogeneity (r^2^ = 0.97, P<0.001, Figure S3a) and also exhibits strong partial correlation with beta diversity holding flow heterogeneity (*SD*_Rxyz_) constant (partial R = −0.99, P = 0.001). Similarly, the standard deviation of *v*_f-indDOC_, an additional descriptor for resource use diversity, correlated with beta diversity (r^2^ = 0.83, P = 0.011, partial R = −0.97, P = 0.006, Figure S3b), but not with flow heterogeneity (r^2^ = 0.47, P = 0.13). Neither gamma diversity nor gamma evenness explained the diversity in resource use.

**Figure 6:**
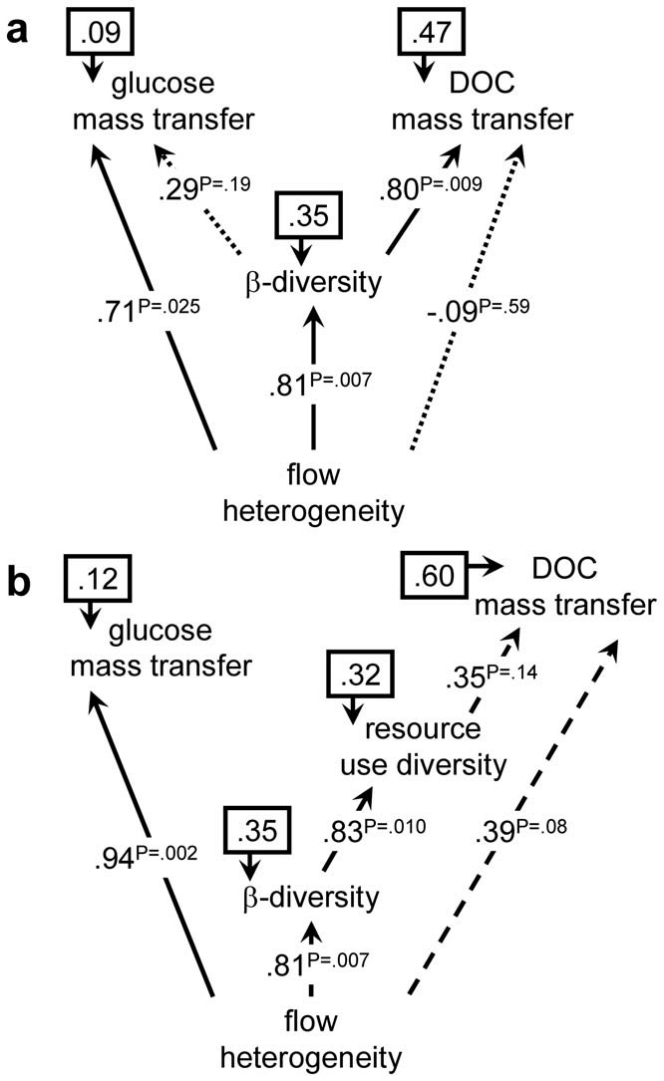
Metavariable path diagrams. Path coefficients were calculated from Mantel statistics (Table 1). Diagram (**a**) and (**b**) with and without resource use diversity, respectively. Line style indicates significance of link. Numbers in boxes represent error variances (%). See Figure S4 for further path diagrams.

To formally test biodiversity and community composition as explanatory links between flow heterogeneity and resource use, we applied causal modeling on dissimilarity matrices (Methods, Table S3) and computed path coefficients from the Mantel correlation structure (Table 1, Figure 6). Glucose uptake seemed to be predominantly controlled by flow heterogeneity. In contrast, path coefficients distributed effects on DOC mass transfer among resource use diversity and flow heterogeneity, which suggests combined controls of flow-induced mass transfer and biodiversity on the use of complex resources. Mantel and partial Mantel tests controlling for the effect of flow heterogeneity confirmed the apparent link between the diversity of DOC use and bacterial beta diversity, the latter being a function of flow heterogeneity (Table 1, Figure 6). The spatial variation of microbial biomass (structural diversity) generated similar patterns (Figure S4, Table S7). Notably, beta diversity strongly correlated with patterns of resource use, which is expressed based on compound-specific mass transfer coefficients and is hence concentration-independent. As a flux, bulk DOC mass transfer depends on both mass transfer coefficients and the skewed distribution of compound concentrations. Hence, resource use distribution and diversity will only partially explain bulk DOC mass transfer.

**Table 1:** Mantel statistics between metavariable dissimilarity matrices.

	β-diversity	resource use distribution	resource use diversity	DOC mass transfer	glucose mass transfer	community composition
**flow heterogeneity**	.81* P = .007	.38 P = .22	**.48 P = .046**	**.55 P = .018**	.94* P = .000	.52 P = .07
**beta diversity**		**.61 P = .011**	.83* P = .008	**.73 P = .010**	.86* P = .004	.50 P = .06
**resource use distribution**	**.55 P = .038**		**.51 P = .017**	.21 P = .23	.29 P = .18	.23 P = .31
**resource use diversity**	.85* P = .007	.40 P = .09		.54 P = .07	**.59 P = .024**	.16 P = .26
**DOC mass transfer**	.57 P = .05	.01 P = .37	.37 P = .12		.67* P = .007	.68* P = .000
**glucose mass transfer**	**.50 P = .047**	−.21 P = .75	.45 P = .09	.53 P = .07		.47 P = .10
**community composition**	.15 P = .33	.04 P = 41	−.12 P = .64	.55* P = .001	−06 P = 49	

Lower diagonal gives partial Mantel statistics (controlling for flow heterogeneity). Significant values are in bold and marked with an asterisk when significant at P<0.05 after Bonferroni-correction. Used dissimilarity matrices: HET (flow heterogeneity), BETA (beta diversity), DOCUSE (resource use distribution), DOCDIV (resource use diversity), DOCMT (DOC mass transfer), GLCMT (glucose mass transfer). See Methods and Table S3 for details of computation.

## Discussion

In this study, we present evidence that the manipulation of fine-scale flow heterogeneity may affect bacterial biodiversity in benthic biofilms and their resource use. The more heterogeneous flow landscapes triggered larger bacterial biodiversity and structural differentiation of biofilms, ultimately resulting in a broader range of DOC compounds removed more rapidly by the biofilms. To our knowledge, this is the first study attempting to integrate microbial diversity with environmental heterogeneity and chemical diversity of DOC as the major resource for microbial heterotrophs.

Ecological theory [7], [48] predicts that biodiversity influences ecosystem functioning through niche differentiation or facilitation, often subsumed under the term “complementarity effect”, and through stochastic processes involved in community assembly, usually referred to as “sampling” or “selection” effects. Combinatorial experimental design and appropriate analysis are required to partition complementarity and selection effects [49]. This works for artificially assembled communities in plots, pots or vials under homogenous and reproducible conditions. It is not a trivial task, however, to manipulate microbial diversity in systems that reflect environmental heterogeneity as in nature. We allowed biofilms to assemble in heterogeneous landscapes from a same natural species pool and at scales relevant to ecosystems. This does not allow for the traditional combinatorial design [49], but our study still informs on the effects of environmental heterogeneity on microbial biodiversity and resource use.

Our results from causal modelling indicate that complementarity as a mechanism underlying the relationship between biodiversity and resource use may be true for DOC as a complex resource but most likely not for glucose. Rather, we argue that glucose uptake is largely controlled by variables modulated by flow heterogeneity, such as turbulence [50] or boundary layer thickness ([28], Singer et al. unpublished data), for instance. Glucose is, in fact, readily available to most microbial heterotrophs for metabolism and biosynthesis. However, the rate-limiting step for glucose uptake can shift from its intrinsic biological availability to limitation of mass transfer through the diffusional boundary layer overlying the biofilm and through resistance to mass transfer within the biofilm – both controlled by hydrodynamics and biofilm architecture [28]. Lower boundary layer thickness in faster or more turbulent flow nonlinearly affects mass transfer to the biofilm [51]. Such hydrodynamic control on mass transfer is supported by the close similarity of glucose mass transfer for different levels of biofilm biomass (Fig. 4b). We therefore suggest flow-induced enhanced mass transfer along increasingly higher bedforms as the prime mechanism underlying the increased glucose uptake in heterogeneous landscapes with more turbulence. Hydrodynamic theory [52] predicts momentum flux and pressure gradients to increase along bedforms as used in our mesocosms, and to generate microhabitats differing in flow velocity, turbulence and mass transfer.

In contrast, we postulate that DOC removal was most likely controlled by mechanisms related to biodiversity rather than just to flow. Several lines of evidence support this hypothesis. The increase of bacterial beta diversity across the gradient of flow heterogeneity suggests diversification of hydrodynamic microhabitats along bedforms to generate distinct local communities assembled from the same species pool in the streamwater [53]. Though our results on DOC uptake and its molecular composition do suggest complementary resource use, Mantel statistics related to gamma diversity and gamma evenness were not significant (Table S7). We recognize that gamma biodiversity does not support complementarity (as niche differentiation) at the level of taxonomic resolution available by T-RFLP. However, we argue that flow heterogeneity generates a small but relevant beta diversity component (at the meter-scale), when biofilms differentiate into functionally non-redundant local communities whose diversified metabolic capabilities may induce regional complementarity [54] along the bedforms. Architectural differentiation and various microbial taxa coexisting in close proximity in stream biofilms [55] may foster facilitation in biofilms. For instance, exploitation and syntrophy [56], [57] or priming by algae [58] (themselves explicitly distributed in space) may help degrade complex molecules. These processes are certainly assisted by small-scale spatial variations of biofilm architecture, related mass transfer and induced resource gradients within biofilms [28]. Ultimately, this may diversify the relative distribution of DOC moieties and shift consumption towards hitherto less attractive DOC compounds. Community composition was also found to be related to DOC mass transfer but not to flow heterogeneity nor to resource use diversity, which suggests regional “selection” [2], [48] at flume-scale as a further mechanism linking biofilm biodiversity to resource use. This effect seems limited, however, as community composition was only weakly influenced by heterogeneity and beta diversity. The “selection” effect could also occur locally, which, according to theory [7], [59], can still be interpreted as (regional) complementarity in heterogeneous environments.

Most studies linking biodiversity to resource use do not account for the molecular diversity of the resource, which, however, can greatly exceed the biodiversity itself [22], [23]. Results from FTICR-MS showed that biofilms in the more heterogeneous landscapes removed a wider range of DOC compounds from the stream water – a pattern that clearly parallels the gradient of bulk DOC uptake. The results also suggest that DOC consumption became increasingly decoupled from compound-specific availability and chemical characteristics with increasing flow heterogeneity. Essentially, this is supported by compound-specific mass transfer coefficients (*v*_f-indDOC_ of 361 compounds) exhibiting less variation at higher flow heterogeneity, which indicates more even (i.e., uniformly distributed) consumption across all compounds of the DOC pool. This would translate into decreasing metabolic specialization as flow heterogeneity increases. Evenness patterns of the molecular composition of both the inflow and residual DOC pools (Fig. 2c) indicate that resource use was limited to the more abundant compounds in less heterogeneous flow, while it gradually included less abundant compounds as flow heterogeneity increased.

Our FTICR-MS results also suggest that more oxygenated compounds were removed more readily from the water. This finding contradicts traditional energetic considerations from microbial food webs models [60]. While process design in biotechnology often rests on the relationship between microbial yield and the degree of reduction of the substrates, microbial ecologists have not used this relationship frequently, at least in part, because of the difficulty of measuring the elemental composition of DOC [60]. Studies that apply ultrahigh-resolution techniques, such as FTICR-MS, to streamwater [24], [25] and groundwater [61] DOC increasingly document the bioreactivity of oxygenated compounds and corroborate our findings. For instance, Kim et al. [58] studied the fate and transformation of DOC on passage through bioreactors containing streambed microorganisms from a temperate and a tropical stream. They found that oxygen-rich molecules were selectively removed and that microbial metabolism modified DOC to lower molecular weight compounds in both streams. Using electrospray-ionization mass spectrometry, Seitzinger et al. [24] found good consistency in the molecular composition of DOC in two streams and a high degree of similarity of compound removal as determined from batch assays from both study streams. Our results complement these previous studies on streamwater DOC molecular structure [24], [25] and findings relating biofilm metabolism to physical habitat heterogeneity [62].

Patterns of bacterial abundance across the six heterogeneity landscapes did not parallel patterns of glucose and DOC removal, indicating no role of bacterial abundance as a driver of resource use. We did not quantify the architectural differentiation of biofilms across flumes, but it becomes obvious from the spatial variation of both chlorophyll-*a* and bacterial abundance among microhabitats, that biofilm differentiation was highest in the more heterogeneous flumes. This is also supported by microscopic analysis of the biofilms and points to an adaptive response of biofilms to the prevailing hydrodynamics in the various microhabitats. For instance, biofilms can exhibit a high degree of architectural differentiation (e.g., floating streamers) enabling them to maximize resource use and to maintain high biomass irrespective of the hydrodynamic conditions [63], [64]. We cannot exclude possible indirect effects of this biofilm differentiation on glucose and DOC uptake. For instance, Bottacin-Busolin et al. [65] showed in the same experimental system that biofilm architectural differentiation as induced by streambed topography increased the transient storage of water and putatively of contained solutes. In line with Battin et al. [28] extended storage of solutes within and around biofilms and hence in close proximity with microbial cells would enhance the uptake of organic compounds.

The observed gradients of mass transfer and resource use diversity were not generated by surface area effects in the flumes. In fact, mass transfer coefficients are expressions of uptake performance corrected for the water volume and streambed surface area in the various flumes. Furthermore, bedforms were sealed to avoid confounding effects from varying streambed topography and induced hydrodynamic exchange and carbon processing [66]. Our experimental design thus mimicked streams with a shallow interfacial zone constrained by bedrock or clay soils [67] or by extensive benthic biofilm growth isolating the deeper sediments in the streambed from the surface water [66].

What are the implications of our study? Streams and rivers are increasingly recognized to contribute to global carbon fluxes, but the underlying mechanisms remain obscure [21]. Our experimental work suggests that diverse (as architectural differentiation and biodiversity) biofilms use a wider range of DOC resources in heterogeneous flow. Underlying candidate mechanisms involve hydrodynamics and related mass transfer, and complementarity potentially induced by microbial facilitation, and selection effects. Thus, complex biophysical mechanisms may underlie the relationship between microbial biodiversity and ecosystem functioning in heterogeneous streams. Our study may thus provide fundamental insights into processes driving globally relevant carbon fluxes in stream ecosystems. Because streams are most vulnerable to human alteration through habitat homogenization [68] and degradation, it is imperative to illuminate these mechanisms at the fine scale.

## Supporting Information

Figure S1 Descriptive statistics of depth and velocity across the flow heterogeneity gradient.
(0.02 MB PDF)

Figure S2 Van Krevelen diagrams of CHONS molecular compositions.
(0.14 MB PDF)

Figure S3 Relationships of beta-diversity with diversity of DOC resource use and bulk DOC uptake.
(0.02 MB PDF)

Figure S4 Alternative metavariable path diagrams calculated from Mantel statistics.
(0.03 MB PDF)

Table S1 Characterization of the hydrodynamic conditions over the control streambed and the bedforms of the landscapes increasing in flow heterogeneity.
(0.04 MB PDF)

Table S2 Water-related environmental background conditions (nutrient concentrations, DOC concentrations and temperature) measured at the outlet of mesocosms and in the header tank.
(0.01 MB PDF)

Table S3 Metavariable dissimilarity (distance) matrices and underlying variables.
(0.02 MB PDF)

Table S4 Results of hierarchical partitioning analysis of DOC compound-specific characteristics as predictors for mass transfer to the streambed.
(0.01 MB PDF)

Table S5 Selected multiple linear regression models using DOC compound-specific characteristics as predictors for mass transfer to the streambed.
(0.01 MB PDF)

Table S6 Results of flume level (gamma) biodiversity partitioning into independent orthogonal components alpha and beta, or richness and evenness.
(0.02 MB PDF)

Table S7 Associations between metavariable dissimilarity matrices as expressed by Mantel statistics.
(0.02 MB PDF)

Text S1 Method Details
(0.11 MB PDF)
