# Supplementary material for: Physical Heterogeneity Increases Biofilm Resource Use and Its Molecular Diversity in Stream Mesocosms
Source: PLoS One. 2010 Apr 1;5(4):e9988. doi: 10.1371/journal.pone.0009988 (PMC2848676; doi:10.1371/journal.pone.0009988)
Supplement: Figure S1 — Descriptive statistics of depth and velocity across the flow heterogeneity gradient. (0.02 MB PDF) [file pone.0009988.s001.pdf]

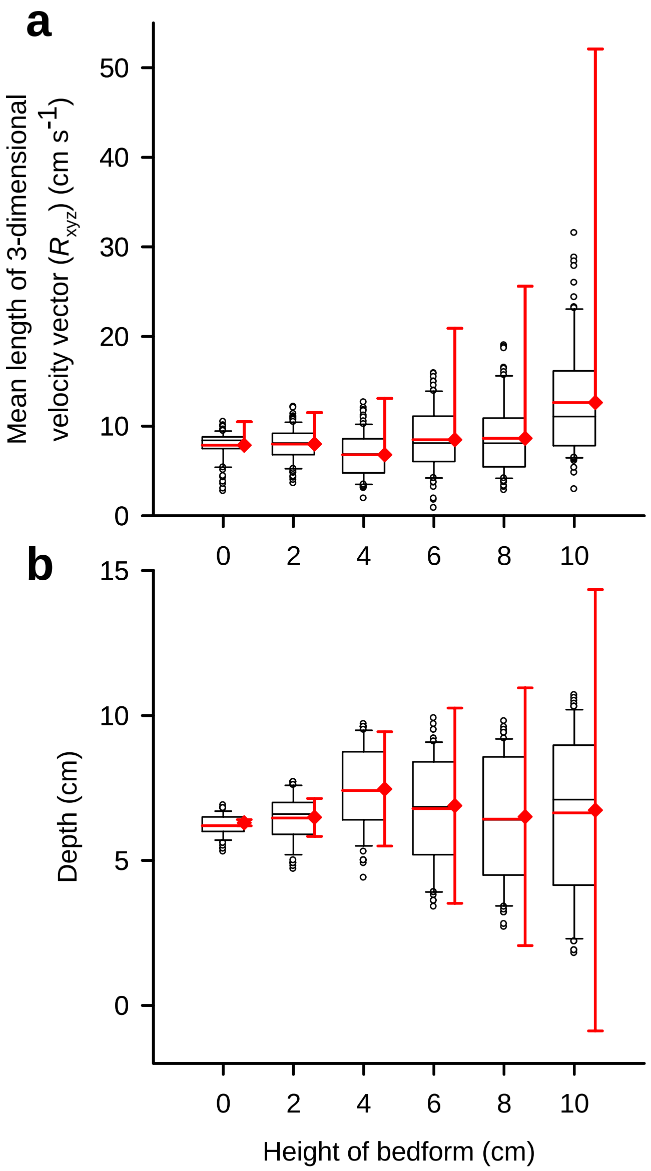

Figure S1. Descriptive statistics of depth and velocity across the flow heterogeneity gradient. Bedforms of 6 different heights create landscapes of increasing flow heterogeneity, i.e. spatial variance of environmental key variables describing hydrodynamic conditions, while holding mean conditions constant across the gradient. **(a)** Height of bedform versus mean length of 3-dimensional velocity vector (R_{xyz}), and **(b)** height of bedform versus depth above bedform. Black lines represent median, boxes delimit 25 and 75 % percentiles, whiskers give 10 and 90 % percentiles, dots are measurements outside the central 90 % region; red line and error bar represent arithmetic mean and variance (a) or standard deviation (b); sample size n=80 measurements distributed regularly over 1 entire bedform of 1 m (each a time series of 3000 data points in case of velocity) per flume.
