# Supplementary material for: Physical Heterogeneity Increases Biofilm Resource Use and Its Molecular Diversity in Stream Mesocosms
Source: PLoS One. 2010 Apr 1;5(4):e9988. doi: 10.1371/journal.pone.0009988 (PMC2848676; doi:10.1371/journal.pone.0009988)
Supplement: Figure S2 — Van Krevelen diagrams of CHONS molecular compositions. (0.14 MB PDF) [file pone.0009988.s002.pdf]

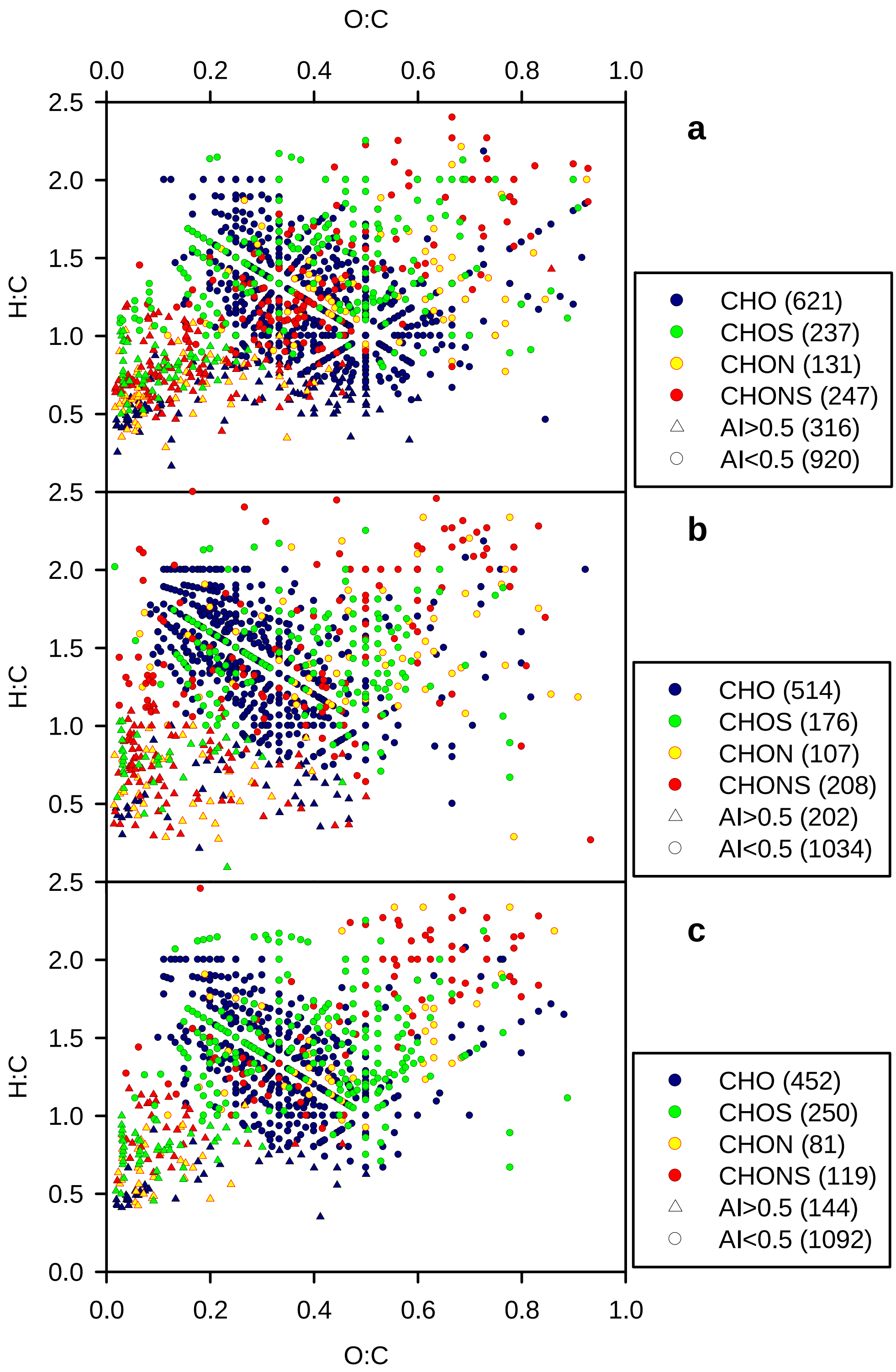

Figure S2. Van Krevelen diagrams of CHONS molecular compositions. (a) inflow DOC pool, **(b)** residual DOC pool in control flume without bedforms, **(c)** residual DOC pool in flume with highest bedforms (10cm). Colours indicate elemental composition, symbol type indicates presence of aromatic structures (aromaticity index > 0.5), values in parentheses indicate number of compounds identified.
