# Supplementary material for: Physical Heterogeneity Increases Biofilm Resource Use and Its Molecular Diversity in Stream Mesocosms
Source: PLoS One. 2010 Apr 1;5(4):e9988. doi: 10.1371/journal.pone.0009988 (PMC2848676; doi:10.1371/journal.pone.0009988)
Supplement: Figure S3 — Relationships of beta-diversity with diversity of DOC resource use and bulk DOC uptake. (0.02 MB PDF) [file pone.0009988.s003.pdf]

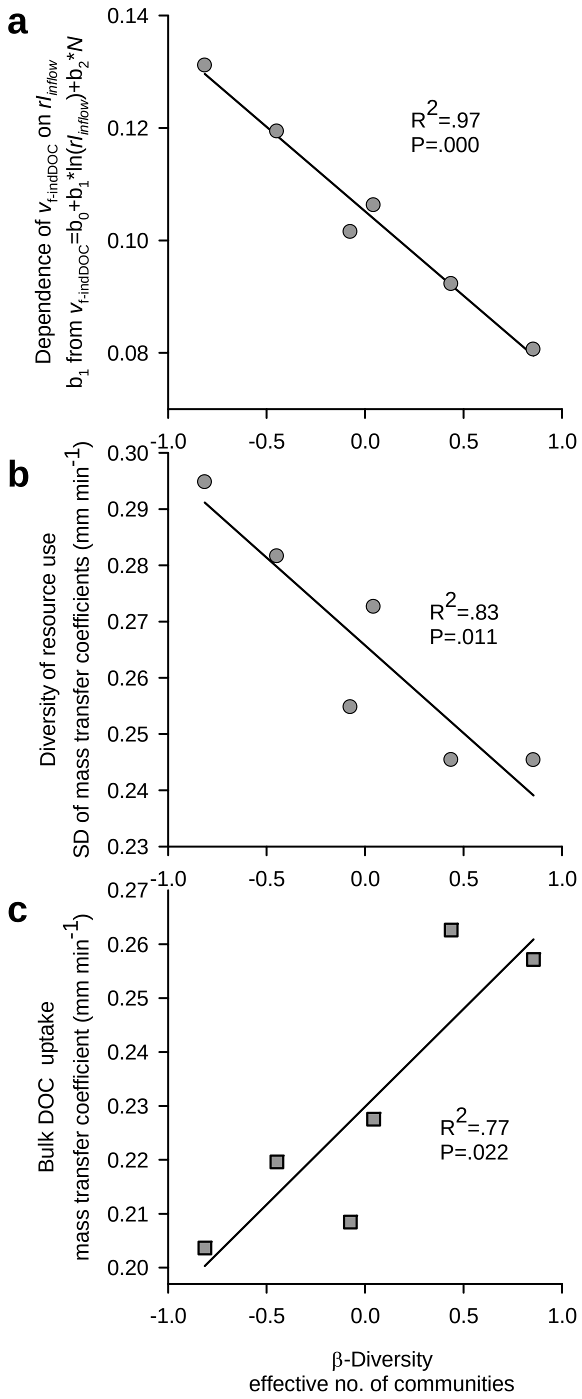

Figure S3. Relationships of β-diversity with diversity of DOC resource use and bulk DOC uptake. Bacterial β-diversity (i.e., the effective number of communities using an Hill exponent q=1) explains (a) the dependence of mass transfer coefficients (mm min-1) on relative availability (i.e., relative peak intensity, rI_{inflow}) in the inflow DOC pool, (b) the diversity of resource use (i.e., the standard deviation of the mass transfer coefficients from 361 individual compounds, and (c) mass transfer coefficient of bulk DOC.
