# Supplementary material for: Physical Heterogeneity Increases Biofilm Resource Use and Its Molecular Diversity in Stream Mesocosms
Source: PLoS One. 2010 Apr 1;5(4):e9988. doi: 10.1371/journal.pone.0009988 (PMC2848676; doi:10.1371/journal.pone.0009988)
Supplement: Figure S4 — Alternative metavariable path diagrams calculated from Mantel statistics. (0.03 MB PDF) [file pone.0009988.s004.pdf]

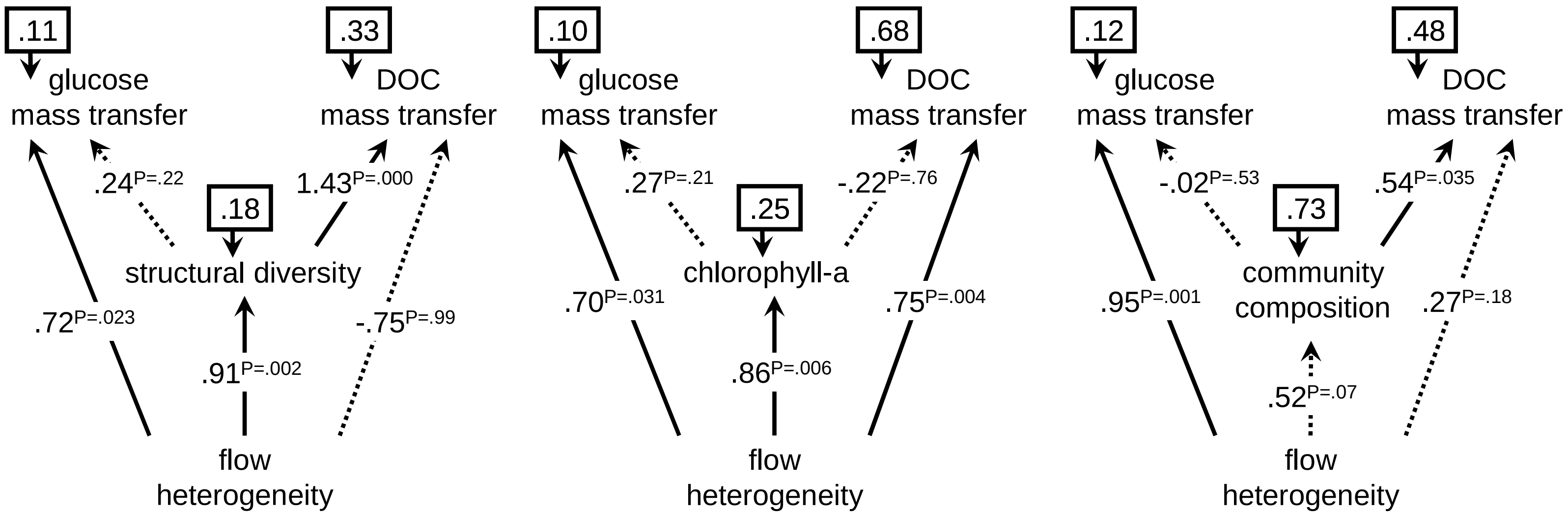

Figure S4. Alternative metavariable path diagrams calculated from Mantel statistics. Besides β-diversity structural diversity, chlorophyll-*a* and community composition exhibited significant Mantel statistics with flow heterogeneity and/or ecosystem processes, and we tested the performance of these variables as explanatory links using path coefficients calculated from Mantel statistics. Line style indicates significance of link. Numbers in boxes represent error variances (%). The following dissimilarity matrices were used: HET (flow heterogeneity), STRUCDIV (biofilm structural diversity), CHLA (chlorophyll-*a*, as a surrogate for autotrophic biomass), COMMCOMP (community composition), DOCMT (DOC mass transfer), GLCMT (glucose mass transfer). See Methods and Supporting Table S3 for details of matrix computations.
