# Supplementary material for: Physical Heterogeneity Increases Biofilm Resource Use and Its Molecular Diversity in Stream Mesocosms
Source: PLoS One. 2010 Apr 1;5(4):e9988. doi: 10.1371/journal.pone.0009988 (PMC2848676; doi:10.1371/journal.pone.0009988)
Supplement: Table S1 — Characterization of the hydrodynamic conditions over the control streambed and the bedforms of the landscapes increasing in flow heterogeneity. (0.04 MB PDF) [file pone.0009988.s005.pdf]

Table S1: Characterization of the hydrodynamic conditions over the control streambed and the bedforms of the landscapes increasing in flow heterogeneity. R_{xyz}… mean length of 3D velocity vector, TKE…turbulent kinetic energy, TI…turbulence intensity; given are mean values ± standard deviation for average and spatial variation of hydrodynamic conditions (n = 80 for each flume, except slope and flume-scale velocity).

	Height of bedforms in the mesocosms – flow heterogeneity treatment					
	no bedforms	2 cm	4 cm	6 cm	8 cm	10 cm
Slope (‰)	0.1	0.1	0.5	1.25	2.0	3.0
R_{xyz} (cm s^{-1})♠	7.9 ± 1.6	8.0 ± 1.9	6.8 ± 2.5	8.5 ± 3.5	8.6 ± 4.1	12.6 ± 6.3
Depth (cm) ♠	-6.3 ± 0.3	-6.5 ± 0.8	-7.5 ± 1.4	-6.9 ± 1.8	-6.5 ± 2.1	-6.7 ± 2.8
TKE (J) ♠	2.0 ± 0.6	2.0 ± 0.9	1.7 ± 1.0	2.7 ± 1.9	2.6 ± 2.0	6.6 ± 7.1
TI (%)♠	41.9 ± 32.5	41.8 ± 38.7	48.6 ± 43.7	50.0 ± 42.7	50.1 ± 44.6	50.2 ± 41.1
Velocity¤	7.63 ± 0.31	7.77 ± 0.36	7.77 ± 0.34	7.53 ± 0.40	7.67 ± 0.38	7.16 ± 0.42

♠ derived from acoustic Doppler velocimetry, n = 80 measurements regularly distributed over one entire bedform.

¤ flume-scale mean velocity calculated from nominal travel time of conservative tracer NaCl, n = 12-16 injections per flume.
