# Supplementary material for: Physical Heterogeneity Increases Biofilm Resource Use and Its Molecular Diversity in Stream Mesocosms
Source: PLoS One. 2010 Apr 1;5(4):e9988. doi: 10.1371/journal.pone.0009988 (PMC2848676; doi:10.1371/journal.pone.0009988)
Supplement: Table S2 — Water-related environmental background conditions (nutrient concentrations, DOC concentrations and temperature) measured at the outlet of mesocosms and in the header tank. (0.01 MB PDF) [file pone.0009988.s006.pdf]

Table S2. Water-related environmental background conditions (nutrient concentrations, DOC concentrations and temperature) measured at the outlet of mesocosms and in the header tank. Given are means ± standard deviation and sample size n. Concentrations of inorganic nutrients and dissolved organic carbon were measured twice a week, temperature was recorded continuously. Concentrations of PO_4-P were mostly below detection limits (< 3 µg l^{-1}) and are not reported.

	Height of bedforms in the mesocosms – heterogeneity treatment						
	header tank	no bedforms	2 cm	4 cm	6 cm	8 cm	10 cm
NO_3-N (mg l^{-1})	**0.57** ± 0.24 (n=22)	**0.53** ± 0.26 (n=17)	**0.54** ± 0.27 (n=17)	**0.51** ± 0.28 (n=17)	**0.52** ± 0.26 (n=17)	**0.51** ± 0.28 (n=17)	**0.53** ± 0.27 (n=17)
NH_4-N (µg l^{-1})	**4.4** ± 4.25 (n=24)	**4.38** ± 4.38 (n=18)	**4.53** ± 5.93 (n=18)	**3.56** ± 3.83 (n=18)	**4.06** ± 5.04 (n=18)	**3.24** ± 3.61 (n=18)	**4.02** ± 4.99 (n=18)
DOC (mg l^{-1})	**1.49** ± 0.52 (n=39)	**1.2** ± 0.37 (n=29)	**1.22** ± 0.35 (n=32)	**1.29** ± 0.35 (n=31)	**1.18** ± 0.34 (n=30)	**1.28** ± 0.38 (n=27)	**1.19** ± 0.36 (n=33)
Temp. (°C)	**9.06** ± 1.31	**9.3** ± 1.37	**9.27** ± 1.34	**9.26** ± 1.36	**9.29** ± 1.34	**9.29** ± 1.35	**9.3** ± 1.35
