# Supplementary material for: Physical Heterogeneity Increases Biofilm Resource Use and Its Molecular Diversity in Stream Mesocosms
Source: PLoS One. 2010 Apr 1;5(4):e9988. doi: 10.1371/journal.pone.0009988 (PMC2848676; doi:10.1371/journal.pone.0009988)
Supplement: Table S3 — Metavariable dissimilarity (distance) matrices and underlying variables. (0.02 MB PDF) [file pone.0009988.s007.pdf]

Table S3. Metavariable dissimilarity (distance) matrices and underlying variables. All matrices are Euclidean distance matrices calculated on standardised variables unless stated otherwise, see Methods for full description of abbreviated variables.

distance matrix	used variables	comments
**HET** (flow heterogeneity)	Standard deviations of *Rxyz*, *depth*, *TKE* and *TI*	Describes similarity in spatial variation of environmental key variables.
**COMMCOMP** (community composition)	Relative peak heights of OTUs from T-RFLP	Bray-Curtis distance matrices for individual sampling dates, rescaled by division through average distance to centroid and averaged to 1 matrix describing similarity in community composition at level of mesocosm (γ).
**BETA** (β-diversity)	Average Bray-Curtis distance among microhabitats within flumes; average effective number of distinct communities for Hill-exponents *q=1* and *q=2*: ${}^{q=1}D_{\beta}$ and ${}^{q=2}D_{\beta}$	3 non-redundant β-diversity measures combined to describe similarity in diversity among microhabitats; i.e. the number of local communities within mesocosms.
**GAMMA** (γ-diversity)	Number equivalents of richness, Shannon-entropy and Gini-Simpson coefficient for whole mesocosms: ${}^{q=0}D_{\gamma}$; ${}^{q=1}D_{\gamma}$; ${}^{q=2}D_{\gamma}$.	3 non-redundant γ-diversity measures combined to describe similarity in diversity at level of mesocosms.
**GAMMA-E** (evenness at γ-level)	Hill-evenness for Hill-exponents *q=1* and *q=2*: ${}^{q=1;0}E$ and ${}^{q=2;0}E$; Shannon-evenness after Pielou (1969): $ln({}^{q=1}D)/ln(S)$	3 non-redundant measures combined to describe similarity in evenness-component of γ-diversity.
**STRUCDIV** (biofilm structural diversity)	Coefficient of variation of bacterial density and chlorophyll-*a* (within flumes)	Describes similarity in spatial variation of biomass-associated structural / micro-architectural variables of biofilms.
**CHLA** (primary producer biomass)	Average chlorophyll-*a* at mesocosm level	Describes similarity in biomass of primary producers and the potential release of exudates as an available substrate for heterotrophs.
**DOCUSE** (DOC resource use distribution)	Normalized mass transfer coefficients of 361 DOC compounds	Describes similarity in consumption patterns of DOC compounds common to all flumes (Figure 2a).
**DOCDIV** (DOC resource use diversity and dependence from constraints)	Diversity of DOC-composition: H'_{DOC}; standard deviation of 361 mass transfer coefficients per flume: SD_{vf}; unstandardised slopes b_1 and b_2 and the coefficient of determination R^2 of a linear regression model predicting v_f from rI_{inflow} and *N*.	Describes similarity in resource use diversity and dependence from controlling factors inflow availability and presence of nitrogen.
**DOCMT** (DOC mass transfer)	Bulk DOC mass transfer coefficients	Describes similarity in bulk DOC dynamics (uptake velocity).
**GLCMT** (glucose mass transfer)	Glucose mass transfer coefficients	Describes similarity in glucose dynamics (uptake velocity).
