# Supplementary material for: Physical Heterogeneity Increases Biofilm Resource Use and Its Molecular Diversity in Stream Mesocosms
Source: PLoS One. 2010 Apr 1;5(4):e9988. doi: 10.1371/journal.pone.0009988 (PMC2848676; doi:10.1371/journal.pone.0009988)
Supplement: Table S4 — Results of hierarchical partitioning analysis of DOC compound-specific characteristics as predictors for mass transfer to the streambed. (0.01 MB PDF) [file pone.0009988.s008.pdf]

Table S4. Results of hierarchical partitioning analysis of DOC compound-specific characteristics as predictors for mass transfer to the streambed. Given are percentage independent contributions applicable to each single variable as predictor of compound-specific mass transfer coefficients (n=276). The sum of independent contributions corresponds to R^2 of the full model (given in the last line). Predictor variables are: ln-transformed relative peak intensity in the inflow (rI_{inflow}), aromaticity index (*AI*), indication of aromatic structures (*AI*>0.5), ratio of oxygen to carbon atoms (*O:C*), ratio of hydrogen to carbon atoms (*H:C*), presence of nitrogen (*N*), molecule size (*m/z*). Significance was tested using 500 random permutations of values of each variable and calculating critical percentiles of distributions of randomized independent contributions (***…P<0.001, **…P<0.01, *…P<0.05, ^{ns}…non-significant). Subsets of predictor variables identified by the Akaike and Schwarz Bayesian Information Criteria to define the most parsimonious model are marked by ^{AIC} and ^{BIC}.

	Height of bedforms in the mesocosms – flow heterogeneity treatment					
	no bedforms	2 cm	4 cm	6 cm	8 cm	10 cm
rI_{inflow}	43.2***^{BIC,AIC}	60.2***^{BIC,AIC}	65.1***^{BIC,AIC}	33.4***^{BIC,AIC}	63.7***^{BIC,AIC}	45.5***^{BIC,AIC}
O:C	30.7***^{BIC,AIC}	17.1***^{BIC,AIC}	12.7***^{BIC,AIC}	30.9***^{BIC,AIC}	10.6**^{BIC,AIC}	22.3***^{BIC,AIC}
H:C	9.1***^{AIC}	8.4**	6.3**	24.9***^{BIC,AIC}	11.1**^{AIC}	18.3***^{BIC,AIC}
AI>0.5	8.4***^{BIC,AIC}	9.1**^{BIC,AIC}	8.1**^{BIC,AIC}	6.8**^{AIC}	11.2**^{BIC,AIC}	10.1**^{AIC}
m/z	5.5**^{AIC}	0.7^{ns}	2.7^{ns}	0.6^{ns}	0.8^{ns}	1.2^{ns}
AI	2.1^{ns}	2.4^{ns}	2.4^{ns}	2.7^{ns}	1.5^{ns}	1.9^{ns}
N	1.1^{ns}	2.2^{ns}	2.7^{ns}	0.7^{ns}	1.1^{ns}	0.7^{ns}
R^2	0.48	0.31	0.38	0.44	0.27	0.26
