# Supplementary material for: Physical Heterogeneity Increases Biofilm Resource Use and Its Molecular Diversity in Stream Mesocosms
Source: PLoS One. 2010 Apr 1;5(4):e9988. doi: 10.1371/journal.pone.0009988 (PMC2848676; doi:10.1371/journal.pone.0009988)
Supplement: Table S5 — Selected multiple linear regression models using DOC compound-specific characteristics as predictors for mass transfer to the streambed. (0.01 MB PDF) [file pone.0009988.s009.pdf]

Table S5. Selected multiple linear regression models using DOC compound-specific characteristics as predictors for mass transfer to the streambed. Unstandardised slopes for predictor variables in 2 multiple linear regression models explaining compound-specific mass transfer coefficients (n=276, m min^{-1}); first model with variables as identified by Akaike Information Criterion (AIC), second model with reduced subset of predictor variables best explaining the flow heterogeneity gradient. The difference between least square (predicted) means at covariate means (predicted effect size) is given for the categorical variables *AI>0.5* and *N* instead of the unstandardised slope. Last column headed R(X, SD_{Rxyz}) states Pearson´s correlation coefficient of unstandardised slopes (or difference between predicted means) with flow heterogeneity (SD_{Rxyz}). Symbols for (1-sided) significance values as follows: ***…P<0.001, **…P<0.01, *…P<0.05, ^{ns}…non-significant.

	Height of bedforms in the mesocosms – flow heterogeneity treatment						
	no bedforms	2 cm	4 cm	6 cm	8 cm	10 cm	R(X, SD_{Rxyz})
models with predictors identified by AIC							
rI	$9.94 \cdot 10^{-5}$ ***	$8.56 \cdot 10^{-5}$ ***	$1.05 \cdot 10^{-4}$ ***	$7.43 \cdot 10^{-5}$ ***	$7.90 \cdot 10^{-5}$ ***	$6.11 \cdot 10^{-5}$ ***	-0.84 *
O:C	$8.65 \cdot 10^{-4}$ ***	$4.26 \cdot 10^{-4}$ ***	$4.16 \cdot 10^{-4}$ ***	$7.65 \cdot 10^{-4}$ ***	$2.60 \cdot 10^{-4}$ *	$3.99 \cdot 10^{-4}$ ***	-0.43 ^{ns}
H:C	$-1.05 \cdot 10^{-4}$ *	$-5.88 \cdot 10^{-5}$ ^{ns}	$-4.55 \cdot 10^{-5}$ ^{ns}	$-2.41 \cdot 10^{-4}$ ***	$-7.45 \cdot 10^{-5}$ ^{ns}	$-1.26 \cdot 10^{-4}$ **	-0.29 ^{ns}
AI>0.5	$2.22 \cdot 10^{-4}$ ***	$1.57 \cdot 10^{-4}$ *	$1.68 \cdot 10^{-4}$ **	$1.17 \cdot 10^{-4}$ ^{ns}	$1.38 \cdot 10^{-4}$ *	$1.15 \cdot 10^{-4}$ ^{ns}	-0.77 *
R^2	0.47	0.31	0.38	0.44	0.26	0.26	-0.61 ^{ns}
reduced models with predictors best explaining the flow heterogeneity gradient							
rI	$1.31 \cdot 10^{-4}$ ***	$1.02 \cdot 10^{-4}$ ***	$1.19 \cdot 10^{-4}$ ***	$1.06 \cdot 10^{-4}$ ***	$9.23 \cdot 10^{-5}$ ***	$8.06 \cdot 10^{-5}$ ***	-0.84 *
N	$4.23 \cdot 10^{-5}$ ^{ns}	$5.28 \cdot 10^{-5}$ ^{ns}	$8.38 \cdot 10^{-5}$ ^{ns}	$2.38 \cdot 10^{-5}$ ^{ns}	$1.15 \cdot 10^{-5}$ ^{ns}	$-1.82 \cdot 10^{-5}$ ^{ns}	-0.84 *
R^2	0.30	0.24	0.32	0.22	0.21	0.16	-0.83 *
