# Supplementary material for: Physical Heterogeneity Increases Biofilm Resource Use and Its Molecular Diversity in Stream Mesocosms
Source: PLoS One. 2010 Apr 1;5(4):e9988. doi: 10.1371/journal.pone.0009988 (PMC2848676; doi:10.1371/journal.pone.0009988)
Supplement: Table S6 — Results of flume level (gamma) biodiversity partitioning into independent orthogonal components alpha and beta, or richness and evenness. (0.02 MB PDF) [file pone.0009988.s010.pdf]

Table S6. Results of flume level (γ) biodiversity partitioning into independent orthogonal components α and β, or richness and evenness. The table gives slopes b_1, coefficients of determination (r^2) and significance levels of linear models of diversity measures on flow heterogeneity (SD_{Rxyz}). γ-, α-, β-diversity and Hill-evenness were computed for Hill exponents q = 1 (i.e., all OTU´s equally weighed by their frequency) and q = 2 (i.e., index is disproportionately sensitive towards common OTU´s). Computations for q = 0 (i.e., index is maximally sensitive towards rare OTU´s) are equivalent to richness. All diversity indices except Pielou´s evenness and mean Bray-Curtis dissimilarity were expressed as numbers equivalents. Full and reduced (red.) set refer to use of all OTUs detected by TRFLP-fingerprinting (full set) or conservative reanalysis using a reduced dataset limited to taxa found at all microhabitats (=samples) at each date which effectively limits the analysis to an investigation of evenness differences. All data standardised and pooled from 4 sampling dates, total sample size = 16 per flume, significant relationships printed bold.

	Hill exponent q = 1<br>Shannon entropy	Hill exponent q = 2<br>Gini-Simpson coefficient
γ-diversity, full set	b_1 = +0.06, r^2 = 0.01<br>P = 0.62	b_1 = +0.12, r^2 = 0.05<br>P = 0.32
γ-diversity, red. set	b_1 = +0.14, r^2 = 0.06<br>P = 0.35	b_1 = +0.13, r^2 = 0.05<br>P = 0.36
α-diversity, full set	b_1 = +0.07, r^2 = 0.01<br>P = 0.57	b_1 = +0.12, r^2 = 0.05<br>P = 0.31
α-diversity, red. set	b_1 = +0.07, r^2 = 0.01<br>P = 0.63	b_1 = +0.08, r^2 = 0.02<br>P = 0.59
β-diversity, full set	**b_1 = +0.31, r^2 = 0.30<br>P < 0.01**	**b_1 = +0.37, r^2 = 0.41<br>P < 0.001**
β-diversity, red. set	**b_1 = +0.45, r^2 = 0.63<br>P < 0.001**	**b_1 = +0.46, r^2 = 0.64<br>P < 0.001**
evenness (Hill) – full set	b_1 = -0.02, r^2 = 0.001<br>P = 0.88	b_1 = -0.06, r^2 = 0.01<br>P = 0.64
evenness (Hill) – red. set	same as γ-diversity	same as γ-diversity
richness (Hill exponent q = 0)	b_1 = +0.04, r^2 = 0.005<br>P = 0.74	
evenness (Pielou) – full set	b_1 = +0.22, r^2 = 0.16<br>P = 0.06	
evenness (Pielou) – red. set	b_1 = +0.14, r^2 = 0.06<br>P = 0.34	
mean Bray-Curtis dissimilarity as β-diversity	**b_1 = +0.41, r^2 = 0.53<br>P < 0.001**	
