# Supplementary material for: Physical Heterogeneity Increases Biofilm Resource Use and Its Molecular Diversity in Stream Mesocosms
Source: PLoS One. 2010 Apr 1;5(4):e9988. doi: 10.1371/journal.pone.0009988 (PMC2848676; doi:10.1371/journal.pone.0009988)
Supplement: Table S7 — Associations between metavariable dissimilarity matrices as expressed by Mantel statistics. (0.02 MB PDF) [file pone.0009988.s011.pdf]

**Table S7. Associations between metavariable dissimilarity matrices as expressed by Mantel statistics.** Mantel (upper diagonal) and partial Mantel (lower diagonal) statistics (controlling for flow heterogeneity), significant values printed bold, P-values not corrected for multiple testing, see Table S3 for variables used for calculation of dissimilarity matrices. Upper left subset of matrix is identical to matrix given in Table 1.

| | **β-diversity** | **resource use distribution** | **resource use diversity** | **DOC mass transfer** | **glucose mass transfer** | **community composition** | **structural diversity** | **Chloro-phyll-*a*** | **γ-diversity** | **γ-evenness** |
|---|---|---|---|---|---|---|---|---|---|---|
| **flow heterogeneity** (HET) | **.81 P=.007** | .38 P=.22 | **.48 P=.046** | **.55 P=.018** | **.94 P=.000** | .52 P=.07 | **.91 P=.000** | **.86 P=.004** | -.28 P=.71 | .09 P=.40 |
| **β-diversity** (BETA) | | **.61 P=.011** | **.83 P=.008** | **.73 P=.010** | **.86 P=.004** | .50 P=.06 | **.74 P=.015** | **.60 P=.032** | -.20 P=.67 | .14 P=.34 |
| **resource use distribution** (DOCUSE) | **.55 P=.038** | | **.51 P=.017** | .21 P=.23 | .29 P=.18 | .23 P=.31 | .24 P=.24 | .00 P=.35 | -.41 P=.98 | .43 P=.16 |
| **resource use diversity** (DOCDIV) | **.85 P=.007** | .40 P=.09 | | .54 P=.07 | **.59 P=.024** | .16 P=.26 | .40 P=.08 | .36 P=.09 | -.25 P=.75 | -.19 P=.73 |
| **DOC mass transfer** (EFDOCMT) | .57 P=.05 | .01 P=.37 | .37 P=.12 | | **.67 P=.007** | **.68 P=.000** | **.75 P=.017** | .42 P=.07 | .26 P=.17 | .15 P=.24 |
| **glucose mass transfer** (EFGLCMT) | **.50 P=.047** | -.21 P=.75 | .45 P=.09 | .53 P=.07 | | .47 P=.11 | **.90 P=.000** | **.88 P=.007** | -.20 P=.59 | -.03 P=.49 |
| **community composition** (COMMCOMP) | .15 P=.33 | .04 P=.41 | -.12 P=.64 | **.55 P=.001** | -.06 P=.49 | | .37 P=.16 | -.52 P=.97 | **.83 P=.049** | .49 P=.16 |
| **structural diversity** (STRUCDIV) | .03 P=.44 | -.25 P=.79 | -.10 P=.59 | **.72 P=.021** | .30 P=.15 | **.60 P=.024** | | **.81 P=.007** | -.12 P=.59 | .18 P=.29 |
| **Chlorophyll-*a*** (CHLA) | -.31 P=.79 | -.69 P=.96 | -.13 P=.63 | -.13 P=.66 | .40 P=.14 | .22 P=.30 | .14 P=.33 | | -.30 P=.80 | -.17 P=.62 |
| **γ-diversity** (GAMMA) | .05 P=.39 | -.34 P=.85 | -.14 P=.63 | **.52 P=.004** | .21 P=.24 | .53 P=.11 | .33 P=.20 | -.11 P=.51 | | .11 P=.41 |
| **γ-evenness** (GAMMA-E) | .11 P=.38 | .43 P=.16 | -.27 P=.81 | .12 P=.28 | -.34 P=.77 | .47 P=.12 | .23 P=.25 | -.50 P=.89 | .14 P=.41 | |
