# Supplementary material for: Physical Heterogeneity Increases Biofilm Resource Use and Its Molecular Diversity in Stream Mesocosms
Source: PLoS One. 2010 Apr 1;5(4):e9988. doi: 10.1371/journal.pone.0009988 (PMC2848676; doi:10.1371/journal.pone.0009988)
Supplement: Text S1 — Method Details (0.11 MB PDF) [file pone.0009988.s012.pdf]

Text S1: Method Details

Flow heterogeneity

The mean length of the 3-dimensional velocity vector $\overline{R}_{xyz}$, the turbulent kinetic energy TKE, and the turbulence intensity TI were calculated according to the following formulas [1,2]:

(i) $R_{xyz} = \sqrt{x^2 + y^2 + z^2}$,

where *x, y* and *z* are velocity components as Cartesian coordinates,

(ii) $TKE = \frac{1}{2}\rho\frac{n-1}{n}\left(SD_x{}^2 + SD_y{}^2 + SD_z{}^2\right)$,

where SD_x, SD_y and SD_z are the respective standard deviations (from n=3000 measurements) of the velocity components and ρ is the density of water, and

(iii) $TI = \frac{SD_{R_{xyz}}}{\overline{R}_{xyz}}$,

where SD_{Rxyz} is the respective standard deviation of the 3-D velocity (from n=3000 measurements). TI is a measure of turbulence standardized for mean velocity, while TKE includes the kinetic energy of mean velocity and turbulence; both describe the fluctuating hydrodynamic environment experienced by the benthic biota. All raw velocity data were automatically processed and subjected to a filtering procedure using a batch-processing code set up in the statistical language R version 2.7.1. [3].

Furthermore, rhodamine injections visualized the flow patterns along the bedforms and particularly the wake-induced turbulence, and flume-scale average velocity was determined weekly from conservative tracer additions.

Biofilm biodiversity: Analysis of bacterial 16S rRNA gene, T-RFLP

DNA from biofilm communities was extracted and purified with the UltraClean Soil DNA Isolation kit (MoBio Carlsbad, Calif.). Non-colonized ceramic coupons served as negative controls. The fluorescently labeled primers used for PCR of the 16S rRNA gene were FAM-labeled 27F and JOE-labeled 1492R (Thermo Electron, Germany) [4]. PCR was performed as described elsewhere [5]. PCR-products were cleaned using gel electrophoresis and the QIAquick Gel Extraction kit (Qiagen). Restriction digests were done as described earlier [5], using approximately 300 ng DNA and the enzyme HhaI. The products were desalted by gel filtration using MultiScreen-HV 96-well plates (Millipore), loaded with Sephadex G-50 (Sigma). The dried product was re-suspended in 10 µl HIDI formamide and 0.5 µl size marker GS2500 Rox (Applied Biosystems), denatured at 95°C and immediately placed on ice. DNA fragments containing the fluorescently labelled forward primer were separated in a Capillary Sequencer 3130 Xl (Applied Biosystems), and electropherograms were analyzed using the GeneMapper software. Restriction fragments smaller than 30bp and larger than 900bp were excluded from further analysis to avoid detection of primers and uncertainties of size determination. Peaks >2% of maximum peak height were clearly distinguishable from background noise. The relative contribution of the respective operational taxonomic units (OTUs) to total community was estimated as peak height divided by the cumulative peak height of the given sample [6,7]; T-RFLP analysis can provide reproducible quantitative results [6,7,8]. Fragments containing the forward and the reverse primer were analyzed separately. T-RFLP patterns produced with the forward primer showed generally more heterogeneity in restriction fragment size than the corresponding T-RFLP patterns containing the reverse primer. All patterns and trends of

community composition and diversity were akin. The results presented in this study therefore refer to the forward fragments because of their higher information content.

Biofilm biodiversity: Rationale and computation details for diversity partitioning and community composition.

The diversity partitioning approach by Jost [9,10] has the following major advantages: (i) the computed β-diversity component is entirely independent of alpha-diversity, (ii) the method yields a continuum of beta diversity measures differing in sensitivity towards rare or common species, and (iii) it integrates a range of popular diversity indices used in general ecology. The key element of this approach is a transformation into effective numbers of species (or number equivalents), that yields a new measure of beta-diversity behaving in agreement with Whittaker´s original definition [11] of beta-diversity. This measure of beta-diversity gives the effective number of distinct communities. Popular measures of similarity and overlap such as the Jaccard, Sorensen, Horn and Morisita-Horn indices are monotonic transformations of this beta-diversity when calculated for two communities (or samples). We have used this approach on TRFLP-data (i.e., relative abundances of operational taxonomic units) with the aim to express average diversity within microhabitats (α) and among microhabitats (β) within each flume. Microhabitats (with local communities) refer to distinct positions along bedforms (section 1.3, Fig. S1a) with characteristic hydrodynamic conditions. While α is a measure for average diversity within local communities, β describes how much local communities diverge from each other. Briefly, computations were done as follows:

When richness, the Shannon entropy and the Gini-Simpson coefficient are expressed in terms of their number equivalents (i.e., the effective number of equally likely elements), they can be expressed in a single formula [9,10,12]:

(iv) $${}^{q}D = \left(\sum_{i=1}^{S} p_i{}^{q} \right)^{1/(1-q)}$$

where *pi* is the proportion of OTU *i* in the sample, *S* is the total number of OTUs, and the exponent *q* determines sensitivity towards rare or common species. At q=0 all OTUs are equally weighted (richness), at q=1 all OTUs are weighted according to their relative frequency (Shannon-entropy), and at q=2 the index is disproportionately sensitive to common species (Gini-Simpson coefficient). Formula (iv) is undefined at q=1, but finds its limit as:

(v) $${}^{q=1}D = \exp\left(-\sum_{i=1}^{S} p_i \ln p_i \right)$$

Expressed as numbers equivalents, regional diversity ${}^{q}D_{\gamma}$ (γ- or flume-level diversity) can be partitioned into 2 independent orthogonal components α- and β- diversity [9]:

(vi) $${}^{q}D_{\alpha} \cdot {}^{q}D_{\beta} = {}^{q}D_{\gamma}$$

with ${}^{q}D_{\alpha}$ being the local diversity (i.e., the effective number of OTUs) and ${}^{q}D_{\beta}$ being a measure for diversity among local communities independent from alpha diversity (i.e., the effective number of distinct communities).

For each flume we calculated ${}^{q}D_{\alpha}$ as the average local diversity:

(vii) $${}^{q}D_{\alpha} = \left(\sum_{j=1}^{N} \frac{1}{N} \sum_{i=1}^{S} pij^{q} \right)^{1/(1-q)},$$

where *pi* is the proportion of OTU *i* in sample *j*, and ${}^{q}D_{\gamma}$ as the regional diversity:

(viii) $${}^{q}D_{\gamma} = \left(\sum_{i=1}^{S} \left(\frac{p_{i1} + p_{i2} + \ldots + p_{iN}}{N} \right)^{q} \right)^{1/(1-q)},$$

where N=4, the number of sampled microhabitats along a bedform. Again, limits for ${}^{q}D_{\alpha}$ and ${}^{q}D_{\gamma}$ exist at q=1 [9]. ${}^{q}D_{\beta}$ is then computed from formula (vi).

Compositional dissimilarity, the Bray Curtis (dis)similarity index, is recommended as a good descriptor of the true resemblance between samples [13,14,15], but not related to any of the above diversity indices [9]. Distances between T-RFLP samples were calculated according to:

(ix) $$d_{ij} = \frac{\sum_{k=1}^{n} \left| x_{ik} - x_{jk} \right|}{\sum_{k=1}^{n} (x_{ik} + x_{jk})},$$

were x_{ik} and x_{jk} are the relative abundances of species *k* in sample *i* and *j* [16]. We used the average Bray-Curtis distance between samples from one flume at a given time as an additional measure for β-diversity.

Regional diversity ${}^{q}D_{\gamma}$ may also be decomposed into the 2 independent orthogonal components richness and evenness [9,10,12,17,18]. Following Hill [12] we calculated evenness as:

(x) $$E_{q,0} = {}^{q}D_{\gamma} / {}^{0}D_{\gamma}$$

for q=1 and q=2, the equivalent evenness expressions for the Shannon entropy and the Gini-Simpson coefficient [17,18]. While $E_{2,0}$ is consistent with the notation of the Simpson evenness [19], $E_{1,0}$ does not agree with Pielou´s classical Shannon evenness definition [20] as $ln({}^{q=1}D)/ln(S)$, which we included in our analyses because of its traditionally widespread use in ecology.

Molecular fingerprinting has limited ability to detect numerically minor taxa [21], and we therefore repeated the analysis using reduced datasets with (i) taxa found in all flumes at γ-level at each date (i.e., at least at one microhabitat), and (ii) taxa found at all microhabitats (=samples) at each date (which effectively limits the analysis to an investigation of evenness differences among widespread and common taxa).

All diversity calculations were done separately for each of 4 sampling dates. To remove temporal variation and to allow a pooled analysis with higher statistical power, data were z-standardized within each sampling date according to:

(xi) $$z_i = \frac{y_i - \bar{y}}{s_y},$$

where y_i is any value of the variable y, $\bar{y}$ is the mean and s_y the standard deviation of the respective variable [15].

To describe similarity among flumes with regard to community composition at flume level (γ) we computed Bray-Curtis dissimilarity matrices between flumes. Flume-level average abundances of OTUs were used to compute separate matrices for each flume and each sampling date. To allow pooling of data with equal weights, distances of each of the date-specific matrices of each flume were rescaled by division through their average distance to the centroid, and rescaled distances were then averaged over all date-specific matrices to form one matrix for each flume (matrix COMMCOMP in Table S3 and Figure 4). Distances to centroids were calculated by principal coordinate analysis as required for objects in a non-Euclidean space [22].

Calculation of mass transfer coefficients

Mass transfer coefficients [23] (uptake velocity v_f, units: length time^{-1}) for glucose and bulk DOC were calculated from log-linear fits of concentration decline in time (t) according to:

(xii) $-\ln(C^*) = k_C \cdot t$

where C^* is the normalized concentration and k_C is the uptake rate coefficient (units: time^{-1}), which is related to v_f by:

(xiii) $v_f = k_C \cdot h = k_C \cdot \frac{V}{A}$

with V being the volume of the system, h being the average depth, and A the streambed area. Mesocosm volumes were estimated at the end of the experiment by injection and dilution of a known amount of conservative tracer (Rhodamin WT). Streambed area was computed from bedform geometry. Time is interaction time with the gravel surface (i.e., corrected for travelling times in the recirculation system).

In contrast to uptake rate or uptake length, the mass transfer coefficient is a measure for uptake which is independent from scaling effects caused by flow velocity and flume dimensions [23,24]. Its product with bulk water concentration gives the flux of a solute to the streambed (units: mass length^{-2} time^{-1}).

DOC-composition: extraction and mass spectrometry details, computation of resource use metrics

2 l water samples collected in the header tank before (inflow) and from each flume after the recirculation period (residual) were filtered through combusted glass fibre filters immediately after collection and slowly passed through a preactivated XAD-8 resin column [25] within hours of collection. The organic fraction retained on the resin was eluted with 50 ml methanol. Methanol was evaporated under vacuum on a Büchi Syncor Polyvap R12 at 30-40 °C and the residue resuspended in 100 µl methanol. This extremely gentle extraction procedure without alkaline/acid treatments was followed to conserve the expected minute compositional differences of DOC along the heterogeneity gradient. Albeit the isolated samples will probably differ from the original DOC due to the loss of a hydrophilic fraction, they can still be expected to be excellent surrogate materials for evaluating DOC compositional changes and bioavailability of various dissolved organic compounds.

ESI(-)-ICR-FT mass spectra were externally calibrated on arginine clusters (10 mg l^{-1} in methanol) and each spectrum underwent systematic internal calibration with fatty acids reaching a mass accuracy lower than 0.1 ppm. Before Fourier transformation of the time-domain transient, a sine apodization was performed. The algorithm used to compute elemental formulae was programmed in Fortran in house. It allows a tolerance of 1 ppm between experimental and theoretical peaks, looks for confirmation by a ^{13}C isotope peak and checks compliance with the nitrogen rule. Further constraints of O/C ratio ≤ 1 and H/C ratio ≤ (2n + 2) were enforced for reliable chemical characterisation. The low signal to noise ratio of 1 allowed consideration of lowest peak intensities for isotopic confirmation, while sensitivity and contamination artefacts were taken care of when data from all samples were conservatively compiled by limitation to compounds positively identified in the inflow and all residual samples (361 individual peaks). This also excludes compounds that were likely produced *in situ* (e.g., photosynthate products from algae) - except there is overlap between *in situ* produced and added compounds. 276 peaks had reliably assigned mass formulae within tolerance limits. Diversity of DOC-composition was calculated using the Shannon-Wiener entropy:

(xiv) $$H'_{DOC} = -\sum p_i \ln(p_i)$$

where p_i is the relative peak height of compounds common to all samples. H'_{DOC} reduces to an evenness measure in this case.

Uptake rates (u) of individual compounds were calculated from peak intensity ratios of inflow and residual for each mesocosm according to:

(xv) $$u_{indDOC-i} = \ln\left(\frac{rI_{resid-i}}{rI_{inflow-i}} \frac{\sum aI_{resid-i}}{\sum aI_{inflow-i}}\right)\frac{1}{t},$$

where $rI_{resid-i}$ and $aI_{resid-i}$, $rI_{inflow-i}$ and $aI_{inflow-i}$ are relative and absolute peak intensities of individual compound i in the residual and inflow DOC-pools, respectively, and t is time of recirculation. A further small correction by mesocosm volume V and area A allows expression as compound-specific mass transfer coefficients:

(xvi) $$v_{f-indDOC-i} = u_{indDOC-i} \frac{V}{A}.$$

Absolute peak intensities are of course unknown, but the quantity $\frac{\sum aI_{resid-i}}{\sum aI_{inflow-i}}$ can be estimated from bulk DOC concentration declines. For further analyses this quantity is of no importance, as it effectively only means an additive transformation of all individual compounds′ v_f ($v_{f-indDOC}$) of a given flume, and will thus not affect model parameters based on the variance of $v_{f-indDOC}$ within each flume. Importantly, statistical independence from bulk DOC mass transfer is uncompromised for both the variance of $v_{f-indDOC}$ and variance-derived model parameters. This variance (or its root, i.e. the standard deviation of mass transfer coefficients) is also used as a measure of resource use diversity across the various DOC compounds. A low variance of $v_{f-indDOC}$ translates into a high similarity of mass transfer coefficients among compounds and means highly even, i.e. diverse, resource use.

Taking the logarithm of the ratio $rI_{resid-i}/rI_{inflow-i}$ further disposes of the problem of choosing between a ratio or its reciprocal, as $\ln(rI_{resid-i}/rI_{inflow-i}) = -\ln(rI_{inflow-i}/rI_{resid-i})$, which makes this quantity (or the related $v_{f-indDOC-i}$) suitable for analyses of similarities of resource use distribution

(i.e., DOC resource consumption patterns) between flumes. This is done in the calculation of a dissimilarity matrix (matrix DOCUSE in Supporting Table S3 and S7 and Figure 4) which is further used for Mantel statistics and can be graphically represented following clustering techniques (Figure 2a). Before calculating dissimilarity measures from $v_{\text{f-indDOC}}$, a normalization (or centering) is a reasonable correction of the additive transformation brought by using bulk DOC concentration declines in formula (xv), which also restores statistical independence from bulk DOC mass transfer.

We analysed dependency of compound-specific mass transfer coefficients from the following chemical descriptors: the ln-transformed relative peak intensity in the inflow rI_{inflow}, the aromaticity index *AI* [26], indication of aromatic structures (*AI*>0.5), *O:C* and *H:C* ratios, presence of nitrogen *N* and molecule size *m/z*. Hierarchical partitioning [27] was applied as the most objective form of multiple linear regression analyses. Hierarchical partitioning overcomes the problems of model selection and of collinearity among predictor variables in multiple linear regression analysis by exhaustive regression model building with all possible variable combinations and partitioning of the coefficient of determination to assign "independent" (as opposed to conjoint) contributions to single predictor variables [27,28]. Significance testing can be achieved by a randomization procedure [28,29]. We used the *hier.part* package [30] in the statistical language R version 2.7.1. [3] and the Akaike and Schwarz Bayesian Information Criteria [31] to select for the most parsimonious model for each mesocosm. This approach identified a subset of predictors repeatedly explaining most of the variance of compound-specific mass transfer in individual flumes. Reduced multiple linear regression models were used to investigate gradients among flumes by relating unstandardised slopes of the regression models to flow heterogeneity (SD_{Rxyz}). Interestingly, *N* as a specific predictor was not identified as a good predictor within flumes, but consistently proved to explain trends across flumes (Supporting Tables S4 and S5). Parameters from a final regression model including rI_{inflow} and *N* as predictors were used to further analyze resource use diversity.

References

1. Bradshaw P (1971) An introduction to turbulence and its measurement. New York, USA: Permagon Press. 232 p.
2. Gordon ND, McMahon TA, Finlayson BL (1992) Stream Hydrology, an Introduction for Ecologists. Chichester, England: John Wiley & Sons Ltd. 526 p.
3. R-Development-Core-Team (2005) R: A language and environment for statistical computing. Vienna, Austria. URL http://www.R-project.org.: R Foundation for Statistical Computing.
4. Lane DJ (1991) 16S/23S rRNA sequencing. In: Stackebrandt E, Goodfellow M, editors. Nucleic acid techniques in bacterial systematics. New York, NY, USA: John Wiley & Sons Inc. pp. 115-176.
5. Moeseneder MM, Winter C, Herndl GJ (2001) Horizontal and vertical complexity of attached and free living bacteria of the eastern Mediterranean Sea, determined by 16S rDNA and16S rRNA fingerprints. Limnology and Oceanography 46: 95-107.
6. Blackwood CB, Hudleston D, Zak DR, Buyer JS (2007) Interpreting ecological diversity indices applied to terminal restriction fragment length polymorphism data: insights from simulated microbial communities. Applied and Environmental Microbiology 73: 5276-5283.
7. Lueders T, Friedrich MW (2003) Evaluation of PCR amplification bias by terminal restriction fragment length polymorphism analysis of small-subunit rRNA and mcrA genes by using defined template mixtures of methanogenic pure cultures and soil DNA extracts. Applied and Environmental Microbiology 69: 320-326.

8. Osborn AM, Moore ERB, Timmins KN (2000) An evaluation of terminal-restriction fragment length polymorphism (T-RFLP) analysis for the study of microbial community structure and dynamics. Environmental Microbiology 2: 39-50.
9. Jost L (2007) Partitioning diversity into independent alpha and beta components. Ecology 88: 2427-2439.
10. Jost L (2006) Entropy and diversity. Oikos 113: 363-375.
11. Whittaker R (1972) Evolution and measurement of species diversity. Taxon 21: 213-251.
12. Hill MO (1973) Diversity and Evenness: A unifying notation and its consequences. Ecology 54: 427-432.
13. Bloom SA (1981) Similarity indices in community studies: potential pitfalls. Marine Ecology Progress Series 5: 125-128.
14. Faith DP, Minchin PR, Belbin L (1987) Compositional dissimilarity as a robust measure of ecological distance. Plant Ecology 69: 57-68.
15. Legendre P, Legendre L (1998) Numerical Ecology. Amsterdam, The Netherlands: Elsevier Science B.V. 853 p.
16. Bray RJ, Curtis JT (1957) An ordination of the upland forest communities of southern Wisconsin. Ecological Monographs 27: 325-349.
17. Buzas M, Hayek L (1996) Biodiversity resolution: an integrated approach. Biodiversity Letters 3: 40-43.
18. Ricotta C (2003) On parametric evenness measures. Journal of Theoretical Biology 222: 189-197.
19. Magurran AE (2004) Measuring Biological Diversity. Oxford, UK: Blackwell Publishing. 256 p.
20. Pielou EC (1969) An introduction to mathematical ecology. New York, USA: Wiley. 286 p.
21. Bent SJ, Forney LJ (2008) The tragedy of the uncommon: understanding limitations in the analysis of microbial diversity. The ISME Journal 2: 689-695.
22. Anderson MJ (2006) Distance-based tests for homogeneity of multivariate dispersions. Biometrics 62: 245-253.
23. Stream, Solute, Workshop (1990) Concepts and methods for assessing solute dynamics in stream ecosystems. Journal of the North American Benthological Society 9: 95-119.
24. Butturini A, Sabater F (1998) Ammonium and phosphate retention in a Mediterranean stream: hydrological versus temperature control. Canadian Journal of Fisheries and Aquatic Sciences 55: 1938-1945.
25. Thurman EM, Malcolm RL (1981) Preparative isolation of aquatic humic substances. Environmental Science & Technology 15: 463-466.
26. Koch BP, Dittmar T (2006) From mass to structure: an aromaticity index for high-resolution mass data of natural organic matter. Rapid communications in mass spectrometry 20: 926-932.
27. Chevan A, Sutherland M (1991) Hierarchical Partitioning. The American Statistician 45: 90-96.
28. Mac Nally R (2002) Multiple regression and inference in ecology and conservation biology: further comments on identifying important predictor variables. Biodiversity and Conservation 11: 1397-1401.
29. Manly BFJ (2006) Randomization, Bootstrap and Monte Carlo Methods in Biology. London, UK: Chapmann & Hall. 455 p.
30. Mac Nally R, Walsh JW (2004) Hierarchical partitioning public domain software. Biodiversity and Conservation 13: 659-660.
31. Quinn GP, Keough MJ (2002) Experimental design and data analysis for biologists. Cambridge, UK: Cambridge University Press. 537 p.
